# Decoding GPCR signaling in living cells to advance early therapeutic discovery for neurological disorders

**DOI:** 10.3389/fnmol.2026.1789400

**Published:** 2026-06-01

**Authors:** Giulia Palladino, Rafael Patarra, Zhenke Li, Moritz J. Rossner, Michael C. Wehr

**Affiliations:** 1 Systasy Bioscience GmbH, Planegg, Germany; 2 Research Group Cell Signalling, Department of Psychiatry and Psychotherapy, LMU University Hospital, LMU Munich, Munich, Germany; 3 Section of Molecular Neurobiology, Department of Psychiatry and Psychotherapy, LMU University Hospital, LMU Munich, Munich, Germany

**Keywords:** barcoded reporters, cell-based assay, GPCR, multiplexed assays, neurodegenerative disorders, neurodevelopmental disorders, psychiatric disorders, safety profiling

## Abstract

G protein-coupled receptors (GPCRs) are major drug targets for neurodegenerative, neurodevelopmental and psychiatric disorders and are targeted by a multitude of marketed drugs. Typically, multiple GPCRs are involved in diseases of this type, making precise modulation of these receptors crucial for beneficial responses in patients. In addition, the regulation of GPCRs by ligands and the concomitant modulation of physiological signaling pathways are highly fine-tuned. Considering these complex roles, the molecular understanding of GPCR biology has advanced considerably in recent years for these disorders. Likewise, recent developments in multiplexed cell-based assays that measure GPCR activities and downstream effects have substantially expanded the tools available for early drug discovery. In this review, we highlight the impact of GPCRs on these complex neurological disorders and review the current state of multiplexed, barcoded assays that can be used to screen for and validate GPCR-modulating compounds in living cells. These multiplexed assays enable rigorous assessment of drug selectivity across on- and off-target profiles, including within closely related GPCR subfamilies, while simultaneously capturing relevant systemic pathway responses. We therefore propose that the widespread use of this technology has the potential to substantially accelerate and de-risk GPCR-targeted drug development.

## Introduction

G protein–coupled receptors (GPCRs) represent the largest and most diverse family of membrane proteins in the human genome, with ~4% of all protein-coding genes encoding GPCRs (Yang et al., 2021). These receptors are integral components of the cell membrane, acting as molecular transducers that convert extracellular signals, such as hormones, neurotransmitters, and sensory stimuli, into intracellular responses (Gurevich and Gurevich, 2017; Zhang et al., 2024). Through this function, GPCRs play crucial roles in regulating a wide range of physiological processes, including metabolism, cell proliferation, differentiation, immune responses, and sensory perception. GPCRs hold extensive pharmacological significance given their wide-ranging involvement in physiological processes and “druggable” structure, i.e., they possess binding sites that are accessible to drugs, and their conformation and activity changes upon drug binding. Around one-third of all marketed drugs (~35%) target GPCRs, yet only about 15% of the human GPCRs are currently exploited as drug targets (Yang et al., 2024). This underscores their therapeutic potential in diverse conditions ranging from cardiovascular and neurological disorders to cancer and inflammation. Despite their dominance as drug targets, further therapeutic exploitation of GPCRs remains limited, largely due to challenges such as receptor redundancy, complex signaling networks, and difficulties in selectively modulating receptor subtypes. Moreover, GPCRs are of major relevance in safety pharmacology profiling, as off-target interactions with non-intended GPCRs can lead to adverse drug reactions (Bowes et al., 2012; Brennan et al., 2024). Thus, systematic GPCR screening remains an essential step in drug development to promote selectivity profiling and safety evaluation.

Structurally, all GPCRs have seven membrane-spanning *α*-helical segments that are connected by alternating intracellular and extracellular loop regions. The N-terminus is located outside the cell, while the C-terminus resides inside the cytoplasm. Based on structural and sequence similarities, GPCRs in vertebrates are often classified into five families: rhodopsin (class A), secretin (class B1), adhesion (class B2), glutamate (class C), and frizzled (class F), according to the GRAFS classification system (Fredriksson et al., 2003). About 90% of all GPCRs belong to the rhodopsin family (Schiöth and Fredriksson, 2005).

Upon ligand binding, GPCRs undergo conformational changes that enable them to interact with various intracellular transducers. The C-terminal tail and the third intracellular loop (ICL3) of the GPCR play a pivotal role in mediating signal transduction downstream of receptor activation. Both structures act as critical structural interfaces for coupling with G proteins and β-arrestins, dictating the specificity, efficacy and diversity of the signaling cascade (Sadler et al., 2023). The conformational flexibility and dynamic equilibrium of ICL3 enable receptors to accommodate different transducers, thereby contributing to biased signaling and pathway selectivity across various physiological contexts. Together with ICL3, the C-terminal tail fine-tunes signaling outcomes by modulating receptor activation, conformation stability, and protein interaction dynamics. However, the highly dynamic nature of these complexes makes the acquisition of well-defined crystallographic structures difficult, thereby limiting the rational design of novel therapeutics targeting GPCR-related disorders (Fouillen et al., 2023) and calling for functional GPCR assays in living cells.

**Figure 1:**
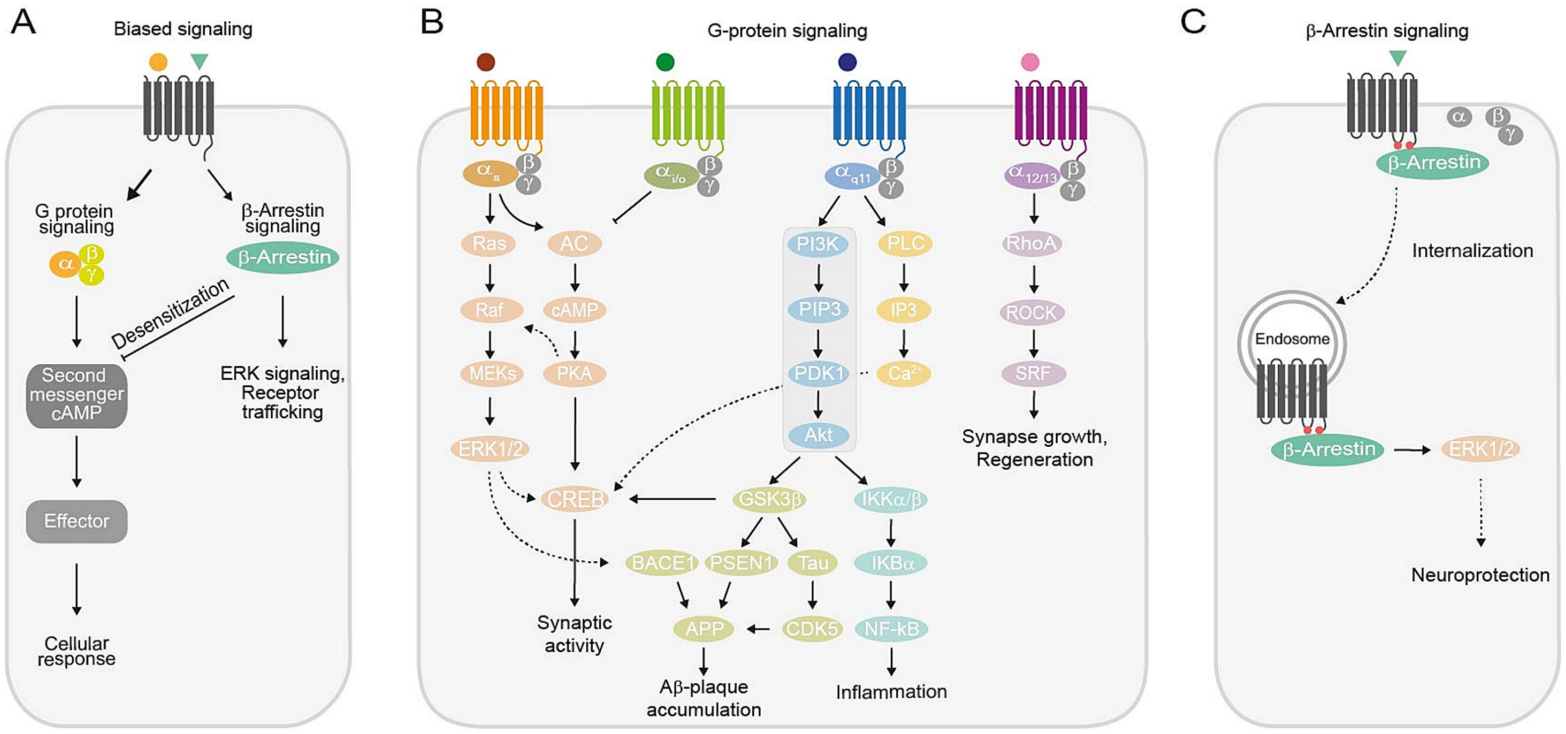
GPCR-mediated signaling pathways. **(A)** Biased signaling of GPCRs. Biased agonists selectively activate either the G protein-dependent (ligand shown as circle) or the β-arrestin-dependent signaling pathway (ligand shown as triangle). G protein-mediated signaling regulates cellular responses primarily through cAMP second messenger activation, whereas β-arrestin-mediated signaling activates the ERK pathway, leading to distinct physiological outcomes. **(B)** Cellular signaling pathways activated by G proteins. Different Gα classes activate distinct GPCR downstream signaling pathways. Gαs activates AC, which increases the production of cAMP. cAMP activates PKA, which phosphorylates the CREB transcription factor. Gαs also modulates the Raf/MEK/ERK signaling pathway (MAPK pathway) that can indirectly influence Aβ accumulation via BACE1. In contrast, Gαi/o inhibits AC and suppresses cAMP-dependent downstream signaling. Gαq/11 activates PLC and stimulates calcium efflux from the endoplasmic reticulum through IP3. Calcium signaling is essential for neurotransmitter release. Gαq/11 also activates PI3K, leading to the production of PIP3 and subsequent Akt (protein kinase B) activation. Akt phosphorylates GSK3β, and a dysregulation of this pathway contributes to tau hyperphosphorylation and Aβ plaque accumulation. Akt also promotes NF-κB activity by facilitating phosphorylation of its inhibitor, IκBα, allowing NF-κB to trigger inflammatory responses that are common and crucial in neurodegenerative diseases. Crosstalk between pathways occurs, for example, through calcium- and GSK3β-dependent modulation of CREB activity. Gα12/13 activates the Rho signaling pathway, leading to RhoA activation, which controls synapse growth, development, and regeneration, linking this pathway to neurological functions and diseases. **(C)** β-arrestin-mediated cellular signaling. β-arrestin binds to the GPCR kinase-phosphorylated intracellular loop 3 and C-terminus of GPCRs (indicated by red dots), leading to β-arrestin-biased signaling, with GPCR internalization and desensitization. β-arrestin promotes anti-apoptotic signals and cell proliferation, modulating the ERK/MAPK signaling. AC, adenylyl cyclase; cAMP, cyclic AMP; PKA, protein kinase A; CREB, cAMP-response element-binding protein; Aβ, amyloid-β; BACE1, beta-site amyloid-β A4 precursor protein-cleaving enzyme 1; PLC, phospholipase C; IP3, inositol 1,4,5-trisphosphate; PI3K, phosphoinositide 3-kinase; PIP3, phosphatidylinositol trisphosphate; GSK3β, glycogen synthase kinase 3β.

Ligands that activate the same GPCR can specifically trigger distinct signaling pathways while avoiding others, a phenomenon known as biased agonism (Figure 1A) (Chundi et al., 2025). Such signaling-biased ligands differentially activate heterotrimeric G proteins and/or induce specific GPCR kinase (GRK)-mediated receptor phosphorylation patterns, leading to specific β-arrestin recruitment and activation of arrestin-dependent signaling pathways, such as ERK signaling (Shukla et al., 2014). This functional specificity arises from ligand-specific stabilization of distinct receptor conformations and coordinates different downstream signaling pathways (Krumm and Roth, 2025). Notably, there are GPCRs that do not signal through G proteins but instead signal exclusively through β-arrestins. These GPCRs, such as atypical chemokine receptors 1 to 4 (ACKR1/2/3/4) carry modifications in the intracellular domains, such as the non-canonical DRY motif, which is critical for G protein coupling, rendering them β-arrestin selective GPCRs (Comerford and McColl, 2024). Furthermore, GPCRs also bind to other transducer proteins, such as Src-family kinases and cyclin-dependent kinase 5 (CDK5) to mediate signaling pathways that are independent of G proteins (Cao et al., 2000; Labus et al., 2021). The β3-adrenergic receptor (ADBR3) binds Src directly (Cao et al., 2000), while the β2-adrenergic receptor (ADBR2) uses a β-arrestin scaffold to interact and promote proliferative MAPK signaling (Pakharukova et al., 2020). HTR7 directly binds CDK5, promoting tau hyperphosphorylation and the formation of tau neurofibrillary tangles, a neuropathological hallmark of Alzheimer’s disease (Labus et al., 2021).

Heterotrimeric G proteins function as molecular switches in GPCR signaling pathways. They are the major class of GPCR signaling transducers, consist of Gα, Gβ, and Gγ subunits (Zhang et al., 2024). There are sixteen Gα subunits that are grouped into four main families: Gαs, *Gαi/o*/z (hereafter called Gαi/o), Gαq/11, and Gα12/13. While each GPCR with Gα coupling capacity exhibits a preference for a Gα subunit, individual receptors can engage multiple Gα subunits with different affinities, enabling diverse and receptor-specific downstream signaling. Upon activation, the Gα subunit undergoes a conformational change that promotes the exchange of guanosine diphosphate (GDP) for guanosine triphosphate (GTP). This nucleotide exchange triggers the dissociation of the Gα–GTP complex from the Gβγ dimer, allowing both components to independently regulate the activity of downstream effector proteins (Young Yang et al., 2024).

Different Gα classes activate distinct downstream signaling pathways (Figure 1B). The Gαs and Gαi subunits regulate adenylyl cyclase (AC) activity, by stimulating or inhibiting it, respectively. AC increases the intracellular production of cyclic AMP (cAMP), which in turn activates the protein kinase A (PKA). Activated PKA directly phosphorylates the cAMP-response element-binding protein (CREB) transcription factor (Wang et al., 2018), whereas the MAPK/ERK pathway (Ras/Raf/MEK/ERK cascade) additionally shapes CREB-dependent transcription trough parallel signalling and pathway crosstalk (Kong et al., 2019). CREB binds to the cAMP-response element (CRE) within target gene promoters to modulate their transcription (Jiang et al., 2022). Gαs and Gαi/o-mediated responses are linked, for example, to dopamine receptor 1 (DRD1) and DRD2 function, respectively, and both receptors have been critically implicated in schizophrenia (Brisch et al., 2014).

Gαq/11 mainly activates phospholipase C (PLC) downstream pathway and signals through calcium (Ca^2+^) to shape, for example, synaptic plasticity (Boczek et al., 2021; Gao et al., 2022). In addition, Gαq/11 modulates PI3K/Akt signaling, which is a crucial intracellular network involved in key processes, particularly withing the CNS, and can affect different downstream signaling pathways. Activated Akt leads to the inhibitory phosphorylation of glycogen synthase kinase-3 beta (GSK3β) and of nuclear factor-κB kinase (IKK), while it activates the mammalian target of rapamycin (mTOR) that inhibits autophagy and induces cell survival (Desale et al., 2021). In Alzheimer’s disease (AD), hyperactive GSK3β drives tau phosphorylation and aggregation as well as presenilin 1 (PSEN1) dependent processing of the amyloid protein precursor (APP) into extracellularly deposited Aβ (Chakraborty et al., 2024). In parallel, beta-site amyloid-β A4 precursor protein-cleaving enzyme 1 (BACE1), the principal β-secretase, cleaves the ectodomain of APP. This process is differentially regulated by ERK1/2, either suppressing BACE1 under oxidative stress (Tamagno et al., 2009), or enhancing it during hypoxia (Yuan et al., 2021). PI3K/Akt is also linked to the inflammatory response in multiple neurodegenerative diseases, such as Parkinson’s disease (PD), AD, and Huntington’s disease (HD). It activates the IKK complex to promote the translocation of nuclear factor kappa B (NF-κB) into the nucleus by inhibiting IκBα (Rai et al., 2019) and driving the expression of proinflammatory genes.

The Gα12/13 family plays critical roles in several physiological processes and cellular functions, including actin cytoskeleton dynamics, mainly through the modulation of RhoGEF-mediated signaling pathways (Suzuki et al., 2009). Rho-ROCK activity downstream of Gα12/13 is associated with synaptic impairment and neuronal loss, as evident in AD, PD, and HD (Wong et al., 2023). Together, the Gα families orchestrate a wide range of cellular responses, with each subfamily involving distinct effectors while contributing to the overall complexity of GPCR signaling.

Different and parallel signaling cascades are triggered by GRKs and β-arrestins, which mainly promote receptor desensitization, internalization and downregulation (Chen and Tesmer, 2022). β-arrestins can also activate MAP kinase (MAPK)/ERK signaling, either at the plasma membrane or at endosomes, to trigger gene activation (Figure 1C) (Kwon et al., 2022; Kahsai et al., 2023). Overall, the complexity of GPCR signaling arises from factors such as differential G protein coupling, biased signaling, pathway crosstalk, and diverse molecular modifications that collectively produce varied and context-dependent signaling outcomes, which are particularly dysregulated in neurological diseases (Saito et al., 2024). Therefore, we first discuss the impact of GPCRs in these disorders, followed by evaluating a methodological approach to analyse activities of multiple GPCRs simultaneously and promote drug development using multiplexed assay techniques.

## GPCRs in neurodegenerative, neurodevelopmental, and psychiatric disorders: mechanistic insights and therapeutic targets

All GPCR classes are present in the brain with diverse roles ranging from neurodevelopment to regulation of neurotransmission, synaptic plasticity, and neuromodulation, converting extracellular signals into intracellular responses. Class A GPCRs are the most abundant type and represent nearly 80% of all brain GPCR transcripts (Uhlén et al., 2015). They mediate most of the fast and modulatory neurotransmission, and the class includes monoaminergic GPCRs (dopamine, serotonin, *α*- and β-adrenergic, and histamine receptors), neuropeptide GPCRs (opioid, cannabinoid, neuropeptide Y, somatostatin, and orexin receptors), and muscarinic acetylcholine receptors. These receptors regulate neuronal excitability and plasticity, for example through DRD1-mediated activation of the cAMP-PKA-CREB pathway, which supports long-term potentiation and memory formation (Zhang et al., 2018). Class B1 is the secretin receptor family and includes the corticotropin-releasing hormone receptor 1 (CRHR1) and the glucagon-like peptide 1 receptor (GLP1R). The main function of this class is to link metabolic and hormonal signaling to brain activity, also playing roles in neuroendocrine regulation and stress response (Reul and Holsboer, 2002; Secher et al., 2014). Class B2 GPCRs, also known as adhesion GPCRs, participate in axon guidance, neuronal migration, mechanosensation, and synaptogenesis, primarily in neurodevelopment but also during synaptic remodeling (Langenhan et al., 2016). Some of the most important elements in Class C in the brain are the metabotropic glutamate receptors (GRM1-8) and *γ*-aminobutyric acid type B (GABA-B) receptors. All these are responsible for the balance between excitatory and inhibitory transmission, modulating plasticity, learning, and memory (Nakanishi, 1994; Mukherjee and Manahan-Vaughan, 2013; Sanchez-Vives et al., 2021). Finally, Class F GPCRs (frizzled and smoothened receptors) serve structural and developmental roles, mediating Wnt and Hedgehog signaling pathways that govern neurodevelopment, synapse formation, and adult neurogenesis (Zheng and Sheng, 2024).

**Figure 2:**
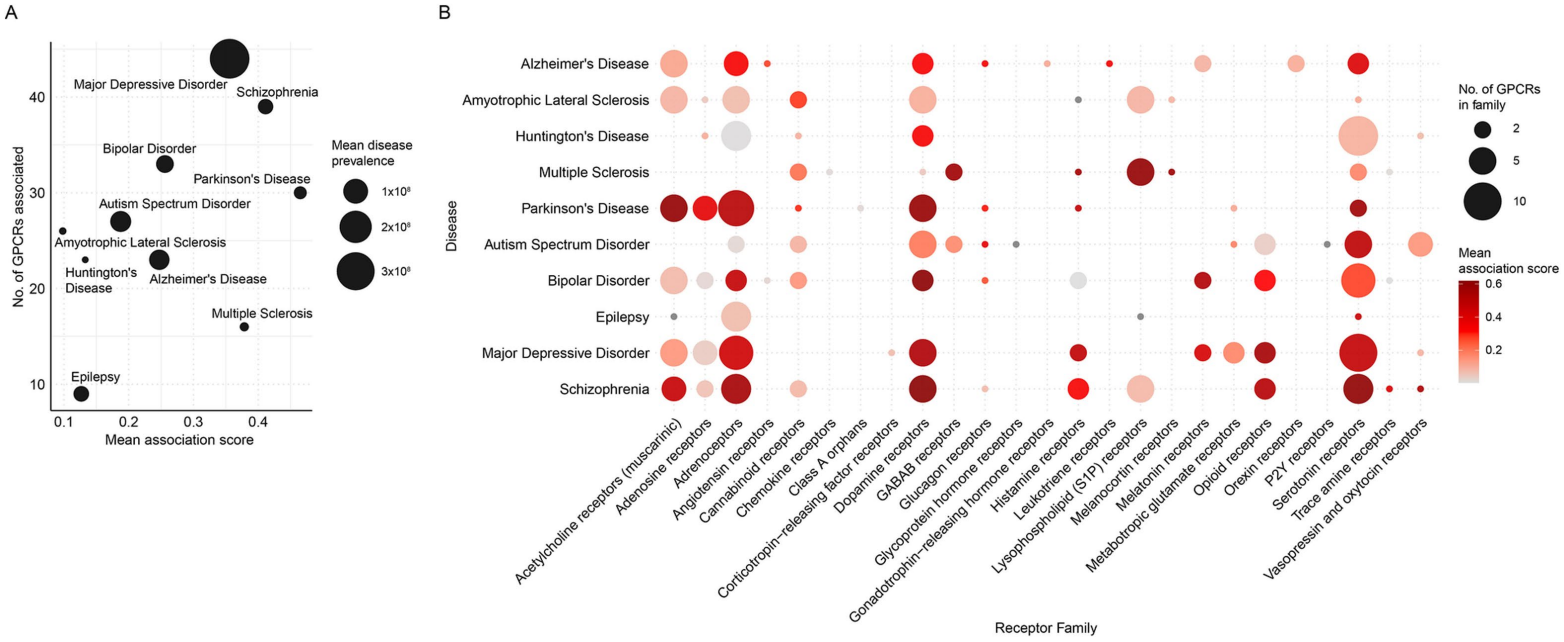
Implications of GPCRs for neurogenerative, neurodevelopmental, and psychiatric disorders. **(A)** Bubble plot shows the relationship between the mean association score (*x*-axis, from 0.1 to >0.4, obtained from GPCRdb) of GPCRs and the number of GPCRs associated (*y*-axis, from 0 to >40) with each disease (Alzheimer’s disease, Parkinson’s disease, Huntington’s disease, Multiple Sclerosis, Amyotrophic lateral sclerosis, Autism, Bipolar disorder, Major Depressive Disorder, Schizophrenia, Epilepsy). The size of the bubbles is proportional to the mean disease prevalence, with larger bubbles indicating higher disease prevalence. **(B)** Bubble heatmap visualizes the relationship between different GPCR families (*x*-axis) and their associations within the neurodegenerative, neurodevelopmental and neuropsychiatric disorders (*y*-axis). Each cell in the grid is represented by a bubble, where the size of the bubble indicates the number of GPCRs (ranging from 1 to 11) in that receptor family associated with a particular disease; the color scale (from grey to dark red) represents the mean association score between the disease and the GPCR family, with darker red indicating a stronger association. Data sources: association scores were extracted from GPCRdb, 2025 (https://gpcrdb.org/), and data for mean disease prevalence were obtained from the World Health Organization (WHO), 2020–2025 (https://www.who.int/), and from the article (GBD 2021 Nervous System Disorders Collaborators, 2024). See Supplementary Table S1 for a detailed description of GPCRs and the score obtained from GPCRdb that associates the targets with the disorders.

Many GPCRs are implicated in neurodevelopmental, neurodegenerative, and psychiatric disorders, reflecting their key roles in disease pathophysiology and symptom management (Wong et al., 2023). Disease-association scoring available in GPCRdb (https://gpcrdb.org) indicates that psychiatric disorders harbor the greatest number and highest scores of implicated GPCRs among these diseases (Figure 2A; Supplementary Table S1). In addition, distinct GPCR subfamilies show preferential involvement across diseases. For example, dopaminergic and serotoninergic receptors are strongly implicated in schizophrenia (SCZ), bipolar disorder (BPD), and major depressive disorder (MDD), whereas lysophospholipid (sphingosine 1-phosphate, S1P) receptors are central to multiple sclerosis (MS) (Figure 2B). This partial disease-specific overlap in GPCR family engagement underscores the need for subtype-selective pharmacological strategies, particularly for multi-target drugs.

In addition, orphan GPCRs, for which no endogenous ligand has been identified yet, have been linked to CNS disorders without a satisfying cure. In neurodegenerative disorders, receptors such as GPR3, GPR6, GPR12, GPR17, GPR26, GPR37, GPR39, GPR55 and GPR78 have been implicated in AD, PD, HD, and MS through roles in neuronal survival, synaptic signaling, neuroinflammation, and myelination (Khan and He, 2017; Kim and Choi, 2024; Öz-Arslan et al., 2024; Lewash et al., 2025). In neurodevelopmental conditions, including autism spectrum disorder (ASD) and epilepsy, altered expression of GPR37, GPR62 has been reported (Rahman et al., 2022; Bolinger et al., 2023; Liu et al., 2025). In neuropsychiatric diseases, receptors such as GPR52, GPR88, and GPR139 are linked to SCZ, BPD, anxiety, and depression (Khan and He, 2017; Azam et al., 2020). While orphan GPCRs represent a compelling target class that would particularly benefit from multiplexed barcoded pathway analysis technologies, a detailed discussion of these receptors falls outside the scope of this review.

In polygenic CNS disorders, pathology spans multiple dysregulated pathways, making multi-target drugs mechanistically necessary, yet indiscriminate receptor modulation risks disrupting the very adaptive signaling they aim to restore. When matched to a patient’s specific receptor or genetic profile, such drugs may further limit off-target exposure, supporting more personalized treatment strategies. Achieving this level of selectivity, however, requires improved assays capable of resolving polypharmacology at the level of individual receptor subtypes (Badria et al., 2025). Dysregulation of GPCR signaling in CNS disorders contributes to subtle alterations in receptor activity, and coupling, trafficking, or downstream pathway balance can lead to substantial network dysfunction. Given this complexity of GPCR biology and the need for more informative drug profiling, we first summarize the overall relevance of GPCR signaling to prevalent CNS disorders. For detailed discussions of the links between GPCR signaling and specific disease mechanisms, we refer readers to comprehensive reviews in the literature (McGregor and Nelson, 2019; Desale et al., 2021; Wong et al., 2023; Marx et al., 2023; Howes et al., 2024; Öz-Arslan et al., 2024; Owen et al., 2025; Annamneedi et al., 2025; Li et al., 2025).

### Alzheimer’s disease

AD is a neurodegenerative disorder that is characterized by progressive impairment of memory, executive functions, and later global cognition. It is the most prevalent cause of dementia and seventh cause of death in the world and predicted to become one of the major public health challenges of this century (Scheltens et al., 2021). The number of AD patients is expected to almost triple by 2050, reaching about 152 million cases worldwide (Nichols et al., 2022). The pathological hallmarks of AD are defined by the formation of neurotoxic β-amyloid plaques as well as the tau neurofibrillary tangles (Madnani, 2023). Despite the undeniable recent progress in biomarker and drug discovery, the high complexity of AD pathways and genetic and cellular heterogeneity between patients continue to pose critical challenges, leaving most patients without effective treatment. At the molecular level, GPCR signaling contributes to AD pathogenesis by modulating amyloidogenic APP processing, tau phosphorylation, neuroinflammatory responses, and synaptic dysfunction.

Several synaptic GPCRs influence amyloidogenic processing of APP. Activation of muscarinic acetylcholine receptors M1 and M3 (CHRM1, CHRM3), the adenosine A2A receptor (ADORA2A), and the *μ*-opioid receptor (OPRM1) upregulate BACE1 expression via PKC–MAPK signaling pathways (Züchner et al., 2004; Xu et al., 2020; Desale et al., 2021). This promotes cleavage of APP into Aβ, which aggregates into soluble oligomers and amyloid plaques that disrupt synaptic function and drive neuroinflammation (Roher et al., 1993). In contrast, activation of the *δ*-opioid receptor (OPRD1) reduces BACE1 levels, highlighting how GPCR signaling bias can differentially regulate Aβ production (Xu et al., 2020).

GPCR-associated signaling components can also regulate tau pathology and cytoskeletal integrity. For example, dysregulation of G protein–coupled receptor kinase 5 (GRK5), which acts downstream of the muscarinic acetylcholine receptor M2 (CHRM2) (Tsuga et al., 1998; Gainetdinov et al., 1999), promotes GSK3β-dependent tau phosphorylation, contributing to the formation of neurofibrillary tangles (Zhao et al., 2019). In addition, the CDK5 can phosphorylate tau and plays a key role in neurofibrillary tangle formation in AD brains (Seo et al., 2017; Ao et al., 2022). CDK5 in turn is activated by the constitutive activity of the serotonin receptor 7 (HT7R) in an G protein-independent manner, suggesting that the HT7R/CDK5 complex acts as critical and integral part of the signaling network involved in the tauopathies, such as AD (Labus et al., 2021; Ackmann et al., 2024).

GPCRs also contribute to neuroinflammatory signaling in AD. Blockade of ADORA2A suppresses production of reactive oxygen species, such as nitric oxide, to provide neuroprotection in the brain (Aires et al., 2019; Dhapola et al., 2025), and also increases APP processing and Aβ secretion in the cerebral cortex of AD transgenic mouse models (Chen et al., 2014).

Finally, in AD, chronic excitotoxicity arises from sustained glutamatergic overactivation and pathological calcium signaling driven by Aβ-mediated synaptic dysfunction. Although excitotoxic damage is primarily mediated by ionotropic glutamate receptors, GPCRs play essential neuromodulatory roles. Metabotropic glutamate receptors are also critically implicated in AD. A notable example is GRM5, which acts as a co-receptor for toxic Aβ42 oligomers through its interaction with the cellular prion protein (PrPᶜ), leading to disruption of normal glutamatergic signaling and synaptic plasticity (Haas and Strittmatter, 2016). In contrast, presynaptic group II (GRM2, GRM3) and group III metabotropic glutamate receptors (GRM4, GRM6, GRM7, GRM8) normally suppress glutamate release and limit excitatory drive (Suh et al., 2018). The dysregulation or loss of these inhibitory GPCR pathways may therefore increase susceptibility to chronic glutamatergic overactivation in AD.

### Parkinson’s disease

PD is a neurodegenerative disorder characterized by motor impairment, including bradykinesia, resting tremor, rigidity, and postural instability, as well as non-motor features such as cognitive impairments and mood disturbances. The pathophysiological hallmark of PD is the selective degeneration of dopaminergic neurons in the substantia nigra pars compacta, leading to dopamine depletion in the striatum. Intraneuronal aggregates composed of misfolded *α*-synuclein, termed Lewy bodies, are a key neuropathological feature and are considered to contribute to synaptic dysfunction and neuronal loss (Marsden, 1990; Ross et al., 2004; Bloem et al., 2021).

Loss of dopamine causes alterations in striatal circuits that lead to imbalances in outputs from the direct and indirect pathways, composed of medium spiny neurons (MSNs) that either express DRD1 receptors or DRD2 receptors, respectively (McGregor and Nelson, 2019). In the healthy state, DRD2 activation triggers Gi/o signaling that reduces cAMP/PKA pathway activity (Surmeier et al., 2007; Kheirbek et al., 2009; Brust et al., 2015). In parallel, it downregulates L-type Ca^2+^ currents via PLC [beta]1-IP3-calcineurin-signaling (Hernandez-Lopez et al., 2000). Finally, DRD2 signaling leads to the opening of G protein-gated inwardly-rectifying potassium (GIRK or Kir3) channels through direct binding of Gβγ subunits and inhibits voltage-gated Ca^2+^ channels such as CACNA1D (Cav1.3) through binding of SHANK1/3 (Olson et al., 2005; Rifkin et al., 2017). These effects ultimately result in decreased neuronal excitability. By contrast in PD, DRD2-mediated inhibition collapses, leading to disinhibited and hyperactive MSNs. The overactivation of the indirect pathway causes excessive thalamic inhibition, and the emergence of bradykinesia and rigidity in patients (McGregor and Nelson, 2019). The DRD2-Gi/o signaling pathway is further antagonized by the interplay between DRD2 and ADORA2A, which is highly co-expressed in the same dendrites of the MSNs and forms heteromers with DRD2. Activated ADORA2A allosterically inhibits DRD2 to increase cAMP/PKA pathway activity through Gαs, interfering with DRD2-associated Gαi/o coupling, further reducing DRD2 signal output and enhanced exacerbated motor control (Prasad et al., 2021). Conversely, ADORA2A antagonists (e.g., caffeine, istradefylline) restore dopaminergic balance by reducing excessive indirect-pathway inhibition and enhancing the effectiveness of DRD2-targeting therapies (Rendón-Ochoa et al., 2022). In contrast to DRD2, DRD1 couples to Gαs activate the cAMP/PKA pathway. Activated PKA also phosphorylates the AMPA receptor (AMPAR) subunit GRIA1 (or GluA1) to promote AMPAR insertion and enhances the activity of CACNA1D (or Cav1.3) L-type Ca^2+^ channels, increasing excitability of direct-pathway MSNs (Surmeier et al., 1995; Mangiavacchi and Wolf, 2004; Gerfen and Surmeier, 2011; Caulfield et al., 2023). DRD1 receptor activation requires high-amplitude phasic dopamine release, which is progressively lost in PD, thereby compromising DRD1-Gαs signaling. According to the acetylcholine/dopamine balance hypothesis, in this low-dopamine state, the increasing cholinergic input to MSNs allows Gαi/o-coupled muscarinic receptor CHRM4 to dominate, reducing PKA signaling and opposing the DRD1-mediated excitation. The resulting shift toward CHRM4 inhibition further weakens direct-pathway output, contributing to impaired movement initiation in advanced PD (Barbeau, 1962; Aosaki et al., 2010). However, in a more recent mouse study, it has been shown that the counterbalance effect of acetylcholine did not come as expected. Instead of an increase in cholinergic signaling after dopamine depletion, the authors verified that, due to the lack of postsynaptic CHRM4, cholinergic signaling was reduced (Nielsen and Ford, 2024).

All these GPCR interactions have guided several therapeutic strategies. For example, antagonists of ADORA2A, such as istradefylline, are clinically used as adjuncts to levodopa, which is a precursor of dopamine capable of crossing the blood–brain barrier to improve motor symptoms (Chen and Cunha, 2020; Kanzato et al., 2024). Furthermore, positive allosteric modulators of DRD1 are in development, and modulation of CHRM4 is being actively explored (Felder et al., 2018; Svensson et al., 2019; Kim and Lee, 2025). In addition to dopaminergic GPCR signaling, other modulatory receptors have been implicated in PD, including GPR55 in striatal motor regulation, HRH4 in microglia-driven neuroinflammation, and metabotropic glutamate receptor 3 (GRM3) that regulates excitotoxicity and indirect-pathway activity (Celorrio et al., 2017; Zhou et al., 2019; Di Menna et al., 2025).

### Huntington’s disease

HD is an autosomal dominant neurodegenerative disorder caused by an expanded CAG repeat in the huntingtin gene. Mutant huntingtin forms misfolded and oligomeric species that disrupt cellular homeostasis and synaptic function, leading to progressive neuronal dysfunction. Neuropathologically, HD is characterized by early degeneration of indirect-pathway MSNs, resulting in progressive striatal atrophy that later extends to cortical regions (Walker, 2007; Tabrizi et al., 2022). Clinically, HD presents motor abnormalities, cognitive decline, and psychiatric symptoms (McColgan and Tabrizi, 2018).

Mutant huntingtin is associated with widespread transcriptional downregulation in striatal neurons, decreasing, for example, BDNF expression. This is accompanied by early reductions in DRD2 receptor levels and altered receptor density at the synapse (Pagano et al., 2016). This uneven loss of dopaminergic receptor signaling produces disorganized dopaminergic modulation and fluctuating dopamine responsiveness, destabilizing inhibitory control over striatal output, and contributing to hyperkinetic motor abnormalities. Consistent with this circuit-level dysfunction, pharmacological reduction of dopaminergic tone can ameliorate chorea during early disease stages (Gibson and Claassen, 2021; Olmedo-Saura et al., 2025). Notably, reductions in both DRD1 and DRD2 are detectable even in presymptomatic individuals, indicating that dopaminergic receptor loss reflects early neuronal impairment rather than secondary degeneration. Moreover, DRD1 and DRD2 level reduction were correlated with both motor and cognitive decline of the patients (Lawrence et al., 1998; Pavese et al., 2003; Pagano et al., 2016).

In parallel, cannabinoid receptor 1 (CNR1) signaling is markedly reduced in the striatum (Laere et al., 2010). Under physiological conditions, MSN-derived endocannabinoids provide retrograde, activity-dependent inhibition of corticostriatal glutamate release. Loss of this excitatory brake enhances glutamatergic drive onto vulnerable MSNs and increases susceptibility to excitotoxic stress (Blázquez et al., 2011). Metabotropic glutamate receptors further modulate excitatory stress in the HD striatum. Postsynaptic GRM5 influences calcium signaling and synaptic plasticity in MSNs and, when activated, has been shown to promote striatal neuron survival in Huntington’s disease models (Doria et al., 2015, 2018). Presynaptic GRM2 and GRM3 act as inhibitory receptors that constrain glutamate release, and their activation in mouse models improves limb coordination (Li et al., 2021). Dysregulation of these receptors, together with loss of CNR1-mediated inhibition, promotes sustained glutamatergic drive and excitotoxic vulnerability in striatal MSNs.

### Multiple sclerosis

MS is a chronic inflammatory disease of the central nervous system. Disease pathogenesis arises from dysregulated interactions between peripheral immune cells, glial populations, and neurons. These interactions drive sustained neuroinflammation ultimately leading to white matter demyelination, axonal damage, and progressive neurodegeneration (Dobson and Giovannoni, 2019). Gray matter involvement and synaptic pathology are also integral components of MS progression, positioning neuromodulatory signaling systems, including GPCRs, as important regulators of disease evolution (van der Poel et al., 2019).

Sphingosine-1-phosphate receptors (S1PRs) play a central role in regulating immune cell trafficking and inflammatory activity associated with white matter pathology in MS and are viewed as key therapy targets (Kandjani et al., 2023; Ran et al., 2025). S1PR1 promotes both acute and chronic neuroinflammation by enhancing microglial and astroglial activation within the CNS, whereas S1PR5 is preferentially expressed in natural killer and dendritic cells and an increase in its expression is critical for their exit from lymphoid tissues, such as lymph nodes and bone marrow (McGinley and Cohen, 2021). Through these receptor-specific actions, S1P signaling governs lymphocyte trafficking across the blood–brain barrier and shapes the magnitude and persistence of immune-mediated demyelination.

Histamine receptors also modulate immune and neural processes in multiple sclerosis. HRH1 and HRH4 promote neuroinflammation by regulating immune cell recruitment and microglial activation, whereas HRH2 exerts immunoregulatory effects that limit excessive inflammatory responses. HRH3, predominantly neuronal, modulates synaptic transmission and network excitability, contributing to gray matter dysfunction (Volonté et al., 2022).

Cannabinoid receptors modulate both neuroinflammatory signaling and synaptic vulnerability in MS. Cannabinoid receptor 2 (CNR2) is predominantly expressed in immune cells and activated microglia, where it regulates cytokine production, immune cell migration, and phagocytic activity, shaping lesion-associated inflammation and limiting immune-mediated damage within the CNS. By contrast, CNR1 is highly expressed at neuronal presynaptic terminals and mediates activity-dependent retrograde suppression of neurotransmitter release, particularly glutamate, providing protection against excitotoxic stress (Nouh et al., 2023). Consistent with these neuromodulatory roles, several cannabinoid receptor agonists have been clinically tested to manage MS symptoms. The most common formulations are tetrahydrocannabinol (THC) and cannabidiol (CBD) combinations and have been proved to be efficient at alleviating spasticity and central neuropathic pain in MS patients (Patti et al., 2016; Sorosina et al., 2018; Turri et al., 2018; Stavrogianni et al., 2025).

Several serotonin receptor subtypes are expressed in immune cells, glia, and neurons, where they influence cytokine release, synaptic transmission, and cortical network activity. In immune cells, HTR1A, HTR2B, and HTR7 signaling is associated with anti-inflammatory and immunoregulatory responses, including promotion of macrophage phenotypes and T-cell programs that limit cytokine-driven inflammation. In contrast, HTR3 signaling has been associated with pro-inflammatory immune activation and may contribute to amplification of autoimmune responses in MS (San Hernandez et al., 2020; Melnikov et al., 2021). Therapeutically, most efforts target the system indirectly via serotonin reuptake inhibitors (SSRIs) to decrease psychiatric symptoms (Stamoula et al., 2021).

GABA-B receptors, which are heterodimers and formed by the two subunits GABBR1 and GABBR2, contribute to MS pathology by regulating inhibitory tone within demyelinated neural circuits and by modulating inflammatory signaling in glial and immune cells. In neurons, GABA-B activation suppresses neurotransmitter release and reduces excitability through presynaptic inhibition of voltage-gated Ca^2+^ channels and postsynaptic GIRK channel activation, counteracting inflammation-associated hyperexcitability in demyelinated networks (Gassmann et al., 2025). Beyond neuronal effects, GABA-B signaling in glia and immune cells modulates Toll-like receptor–dependent inflammatory pathways, influencing cytokine production, and innate immune responses. Accordingly, agonists such as baclofen impact both circuit excitability and neuroinflammatory signaling in MS (Sammaraiee et al., 2019).

In addition, several GPCRs, including the orphan receptor GPR13, have been found to modulate remyelination after injury (McDonald et al., 2025). A phenotypic drug screen identified many existing drugs capable of enhancing oligodendrocyte differentiation, and clinical trials in MS patients have been initiated accordingly (Najm et al., 2015). The most promising candidates are clemastine and benztropine, which target GPCRs of the histamine and muscarinic acetylcholine receptor families (Caprariello and Adams, 2022).

### Amyotrophic lateral sclerosis

Amyotrophic lateral sclerosis (ALS) is a fatal neurodegenerative disorder characterized by the selective and progressive loss of upper and lower motor neurons. In contrast to immune-mediated movement disorders such as MS, ALS is driven by the intrinsic vulnerability of motor neurons to excitatory imbalance and calcium dysregulation, resulting in rapid and irreversible degeneration. This excitatory vulnerability arises from the accumulation of misfolded and aggregated proteins, including TDP-43, SOD1, and FUS, which disrupt RNA metabolism, protein clearance pathways, and synaptic homeostasis (Rosen et al., 1993; Neumann et al., 2006; Kwiatkowski et al., 2009).

In ALS, group I metabotropic glutamate receptors (GRM1 and GRM5), amplify postsynaptic excitatory and calcium-dependent signaling, and exacerbate inflammatory responses in glial cells (Battaglia and Bruno, 2018). GRM1 and GRM5 are upregulated in human ALS patient spinal cord neurons and astrocytes (Aronica et al., 2001). In contrast, group II receptors (GRM2 and GRM3) act as inhibitory regulators of glutamatergic tone, limiting presynaptic glutamate release and promoting astrocyte-mediated neuroprotective responses (Battaglia and Bruno, 2018). Therapeutically, inhibition of GRM5 using a negative allosteric modulator, 2-chloro-4-((2,5-dimethyl-1-(4-(trifluoromethoxy)phenyl)-1H-imidazol-4-yl)ethynyl)pyridine (CTEP), reduces symptoms and increases survival rate in SOD1 G93A ALS mice models (Milanese et al., 2021). In contrast, activation of Group II mGluRs with agonists such as (1R,4R,5S,6R)-4-Amino-2-oxabicyclo[3.1.0]hexane-4,6-dicarboxylic acid (LY379268) reduced the symptoms but did not increase the survival rate of the same SOD1G93A mice model (Corti et al., 2004).

In addition, cannabinoid receptors modulate excitatory and inflammatory stress in ALS. As previously discussed for MS, CNR1 and CNR2, modulate presynaptic glutamate release and regulation of microglial activation, respectively, demonstrating potential for treatment options with THC/CBD to attenuate stressors that exacerbate motor neuron vulnerability in ALS (Lacroix et al., 2023).

### Autism spectrum disorder

ASD is a neurodevelopmental disorder that is diagnosed in 1:100 children worldwide, based on the occurrence of deficits in social interaction and communication as well as repetitive behaviors (Zeidan et al., 2022). Genetically, various copy number variations (CNV), such as duplication and deletion 16p11.2, deletion of 22q11.2, as well as genes associated with synaptic functions, such as SHANK2 and SHANK3, Neuroligin 3 and 4 (NLGN3, NLGN4), and synaptic Ras GTPase activating protein 1 (SYNGAP1), are strongly associated with ASD (Grove et al., 2019; Rylaarsdam and Guemez-Gamboa, 2019; Fu et al., 2022). Dysregulated signaling of GPCRs is also implicated in ASD (Annamneedi et al., 2025). Among these receptors, DRD2, CNR1, the oxytocin receptor (OTXR), and the vasopressin receptor 1A (AVPR1A) are considered key targets for treating ASD. For example, DRD2 expression is increased in autism patients causing changes in the indirect pathway and circuity of MSNs in the striatum, thereby inhibiting competing movements selected by DRD1-controlled direct pathway (Brandenburg et al., 2020). Furthermore, AVPR1A regulates social behaviors and plasma levels of OTXR are reduced in children (Cataldo et al., 2018; John and Jaeggi, 2021). In addition to these receptors, the activity and expression levels of serotonin receptors 2A (HTR2A) (Beversdorf et al., 2012; Oblak et al., 2013; Liu et al., 2021) and 7 (HTR7) (Helsmoortel et al., 2016) are implicated ASD. In particular, HTR7-selective agonists are potential therapeutic options, as they improved social interaction in a mouse model (Khodaverdi et al., 2021). Nevertheless, while HTR7-selective agonists have shown promise in improving social interaction in preclinical models, clinical research in autism has to date focused on broader serotonergic modulation, such as the study investigating acute effects of citalopram, an SSRI, tianeptine, another effective antidepressant but with a different mechanism of action to SSRIs, and placebo on brain response in males with and without autism spectrum disorder (ClinicalTrials.gov Identifier: NCT04145076), rather than on HTR7-specific agents. These findings suggest that dysregulation of specific GPCRs represents a key mechanism underlying ASD and a promising target for therapeutic intervention.

### Major depressive disorder

MDD, also known as depression, is a common psychiatric disorder characterized by persistent sadness and anhedonia, a condition where patients do not feel pleasure from life’s experiences or interest in activities for a long period of time. In addition, patients suffer from cognitive deficits (Marx et al., 2023; Qian et al., 2022). Depression affects approximately 4% of the global population, with an increased rate of 5.7% in adults. Notably, depression is around 1.5 times more prevalent among women (6.9%) than men (4.6%) (Albert, 2015). Beyond emotional and cognitive symptoms, depression is associated with dysregulation of stress-response systems, neuroinflammation, and impaired neuroplasticity, all of which contribute to its chronic and recurrent nature (Cui et al., 2024). Among the first available treatments for depression, there were tricyclic antidepressants, including imipramine, amitriptyline, clomipramine, desipramine, and doxepin (Arroll et al., 2005; Moraczewski et al., 2023). These drugs inhibit the reuptake of mainly serotonin and norepinephrine, but also to small extent dopamine, thereby increasing their concentrations in the synaptic cleft and producing antidepressant effects. Their interaction with additional receptor systems, such as muscarinic acetylcholine receptors, histamine receptor H1 (HRH1), DRD2, HTR2A, and alpha-1 adrenergic receptors (ADRA1A, ADRA1B, ADRA1D), also contributes to their pharmacological and side-effect profiles (Gillman, 2007; Nojimoto et al., 2010; Obara et al., 2024). Novel therapeutic strategies are increasingly exploring GPCR modulation to achieve faster and more robust antidepressant effects. For instance, rapid-acting antidepressants such as ketamine exert part of their downstream effects through GPCR-mediated pathways involving glutamate and G protein signaling. The result is a rapid antidepressant effect that emerges within hours (Krystal et al., 2024). These mechanistic findings are supported by human clinical trial (ClinicalTrials.gov Identifier: NCT03237286) showing that a single intravenous injection of ketamine rapidly reduces depressive symptoms with effects lasting several days. Trials in treatment-resistant patients consistently demonstrate its robust and reproducible efficacy, prompting the development of novel related treatments (Rengasamy et al., 2024).

### Schizophrenia

SCZ is a highly heritable mental disorder affecting approximately 1% of the global population and characterized by positive symptoms (e.g., hallucinations, delusions), negative symptoms (e.g., lack of emotion and motivation and social withdrawal), and cognitive deficits (Falkai et al., 2015; Trubetskoy et al., 2022; Owen et al., 2025). Its origins involve interacting genetic, neurodevelopmental, and environmental factors, with convergent dysregulation of neurotransmitter systems governed largely by GPCRs. These receptors shape neuronal excitability, synaptic integration, and large-scale circuit dynamics, making them central to current pathophysiological models.

The dopamine hypothesis remains foundational, with hyperactivity of DRD2-regulated pathways in subcortical regions contributing to positive symptoms, while reduced dopaminergic signaling at prefrontal DRD1 receptors is associated with cognitive impairment and negative symptoms (Brisch et al., 2014). Typical neuroleptics such as haloperidol and chlorpromazine exert strong DRD2 antagonism, yielding robust antipsychotic effects but also extrapyramidal symptoms. However, because a strong inhibition of DRD2 often produces motor and metabolic side effects, attention has shifted toward more selective GPCR targets and signaling pathways. Atypical agents, including clozapine, risperidone, and olanzapine, combine moderate DRD2 antagonism or partial agonism, as is the case for aripiprazole, with broader GPCR profiles, resulting in improved tolerability and, in some cases, better outcomes for negative and cognitive symptoms (Meltzer and Gadaleta, 2021; Howes et al., 2024).

Serotonergic GPCRs, particularly HTR2A, modulate cortical microcircuit activity and are implicated in perceptual and cognitive disturbances. Hallucinogens that act as HTR2A agonists illustrate how their overactivation can produce psychotomimetic states. Many second-generation antipsychotics, like clozapine, risperidone, and olanzapine, combine DRD2 inhibition with HTR2A antagonism, which is thought to enhance efficacy for negative and cognitive symptoms and mitigate motor side effects (Meltzer and Gadaleta, 2021).

Glutamatergic dysfunction, particularly NMDA receptor (NMDAR) hypofunction, is increasingly recognized as a pathophysiological pathway linking molecular, cellular, and circuit-level abnormalities (Javitt, 2023). GPCRs that potentiate glutamate release through their role in the NDMAR interactome, such as metabotropic glutamate receptors, are therefore attractive targets. Group II metabotropic glutamate receptors (GRM2/GRM3) agonists have shown potential to dampen excessive glutamate transmission and reestablish cortical signaling balance, although clinical trials have yielded mixed findings (Li et al., 2015).

Cholinergic GPCRs have regained prominence with the emergence of selective M1/M4 (CHRM1/CHRM4) modulators. Reductions in M1/M4 signaling correlate with cognitive deficits and altered circuit synchrony. Cobenfy (xanomeline–trospium chloride, initially called KarXT during development and clinical trials), an M1/M4-preferring agonist/peripheral antagonist combination, demonstrated antipsychotic efficacy without blocking DRD2 receptors. It was recently evaluated in phase 3 clinical studies to improve both positive and negative symptoms of schizophrenia, having also an enhanced safety profile, and approved by the Food and Drug Administration (FDA) in September 2024 (Kaul et al., 2024; Kingwell, 2024). However, cobenfy was later shown to have no beneficial effects when applied as add-on therapy, while single application showed improved negative symptoms and potential cognitive benefits (clinical trial ID NCT05145413 at ClinicalTrials.gov). Nevertheless, these findings highlight the therapeutic promise of targeting muscarinic GPCRs. Collectively, these observations depict schizophrenia as a disorder of distributed circuit dysfunction involving multiple GPCR-mediated pathways rather than isolated neurotransmitter abnormalities, making these receptors central targets for more selective therapeutic strategies.

### Bipolar disorder

BPD is a chronic psychiatric disorder affecting approximately 1–2% of the global population, characterized by alternating episodes of mania and depression, often with cognitive and functional impairments. Its pathophysiology involves complex interactions between genetic, neurochemical, and environmental factors, with dysregulation of neurotransmitter systems playing a central role (Oliva et al., 2025; O’Connell et al., 2025). GPCRs, including dopaminergic receptors (DRD1, DRD2), serotonergic receptors (HTR1A, HTR2A, HTR2C), and adrenergic receptors α1 and α2 (ADRA1A/B, ADRA2A) are critically implicated in BPD. For example, ADRA1A and ADRA1B were linked to mood regulation, with ADRA1A being critical for mediating antidepressant effects (Doze et al., 2009), while ADRA2A was recently reported to be implicated for stress-related BPD (Ejiohuo et al., 2025). Altered GPCR function can disrupt intracellular signaling cascades, such as cAMP pathways and PKA activity, contributing to mood instability. In this scenario, an activation of the cAMP-PKA pathway is attributed to a rapid antidepressant effect (Gao et al., 2022).

Notably, the GPCR-mediated dysregulation in BPD shares significant overlap with schizophrenia, particularly in dopaminergic and serotonergic signaling, suggesting convergent molecular mechanisms underlying affective and psychotic symptoms (Owen et al., 2023). Pharmacological interventions, including mood stabilizers and atypical antipsychotics such as quetiapine, olanzapine, risperidone, and aripiprazole, modulate these GPCR pathways, underscoring their therapeutic relevance.

### Epilepsy

Epilepsy is a chronic neurological disorder that is characterized by recurrent, unprovoked seizures arising mainly from an imbalance between excitatory and inhibitory ion channels in the brain. Loss of function in the excitatory channels or gain of functions of the inhibitory channels is also reported in epileptic encephalopathies (Debanne et al., 2024). Epilepsy affects approximately 65 million people globally (Devinsky et al., 2018). Despite the availability or anti-seizure medications, most of which act on ion channels, approximately 30% of patients are affected by non-controlled seizures. This treatment gap highlights the need for alternative therapeutic strategies targeting other mechanisms of neuronal excitability. GPCRs play a critical role in regulating synaptic transmission and maintaining the balance between excitation and inhibition. Disruption of GPCR signaling can therefore contribute directly to seizure generation and propagation. Several GPCRs have recently emerged as promising therapeutic targets in epilepsy (Yu et al., 2019). For example, activation of GPR40 has been shown to reduce epileptic activity in animal models by affecting NMDA receptor function and expression, indicating its potential as a novel therapeutic target (Yang et al., 2018).

The rare forms of epilepsy that affect children, such as Dravet syndrome, Lennox–Gastaut syndrome, and Tuberous Sclerosis Complex, can be treated with CBD as an anti-seizure drug. CBD helps reducing epileptic seizures by targeting both ion channels, by controlling excitability, and the GPR55 receptor, a GPCR activated by lysophosphatidylinositol, an activity-dependent lipid messenger, modifying the excitatory-inhibitory ratio of synapses (Morano et al., 2020; Tsien and Rosenberg, 2025).

Recently, the lactate receptor HCAR1 (GPR81) has been identified as a key modulator of seizure activity. HCAR1 is known to decrease neuronal activity in physiological conditions. Mice deficient in HCAR1 show increased seizure severity and duration compared to wild type mice, highlighting its importance in preventing uncontrolled neuronal activity (Alessandri et al., 2024).

Given the central role of GPCRs in neurodegenerative, neurodevelopmental and psychiatric diseases (De Deurwaerdère et al., 2022), GPCR-targeted mechanisms represent a compelling and underexploited frontier for next-generation therapeutic development, offering pathways beyond the limitations of current treatments. However, realizing this potential requires technologies capable of simultaneously profiling compound activity across multiple GPCRs and their downstream signaling pathways in living cells. Barcoded GPCR assays are particularly well-suited to meet this need, enabling high-throughput, multiplexed assessment of efficacy, potency and off-target liability within a single integrated experimental framework. This streamlines the drug discovery process and improves the translational relevance of early-stage compound profiling.

## Target- and pathway-based screening approaches for accelerating drug discovery

The early phase of drug discovery is a critical step for identifying drug candidates that progress into preclinical testing, clinical trials, and are finally approved for market distribution (Sandrin et al., 2024). This process is particularly challenging for identifying drugs for complex disorders that are caused by the deregulation of multiple targets and interconnected pathways rather than a single target. The development of polypharmacological drugs, i.e., drugs that can selectively target multiple receptors, offers a powerful strategy for treating these multifactorial diseases (Roth et al., 2004). As multiple GPCRs are involved in one single complex disorder (see above), the modulation of various GPCR activities, and their coordinated, network-level activities can be a key part of a therapeutic response. Therefore, integrated target- and pathway-based screening approaches (the latter may also be classified as phenotypic) are needed to understand how a drug modulates the entire GPCR network, with the aim of developing drugs that have the desired effect while minimizing side effects.

**Figure 3:**
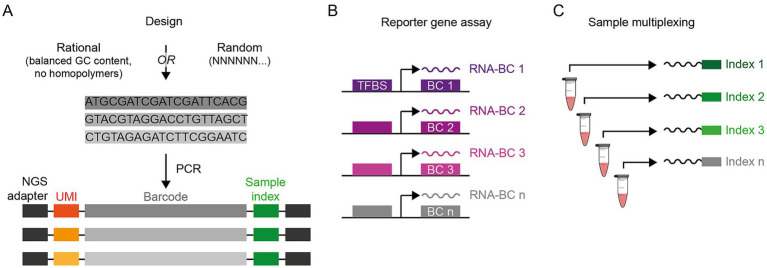
Molecular barcodes for multiplexed cell-based profiling of transcriptional activities. **(A)** Barcode (BC) design: Unique DNA barcode sequences are generated, either rationally (balanced GC content, avoiding homopolymers) or randomly, to label individual reporter constructs. For downstream identification via next-generation sequencing (NGS), an NGS adapter, a unique molecular identifier (UMI), and a sample index are added to each barcode via PCR. **(B)** Application of molecular barcodes in reporter gene assay, acting as indirect readouts of specific reporter activities and enabling simultaneous quantification of multiple targets. **(C)** Molecular barcodes (Index 1, 2, 3, n) enable pooling of multiple samples into a single sequencing run.

To capture a broad spectrum of receptor responses in parallel, barcode-based assays offer a strong framework to accelerate GPCR drug discovery and advance polypharmacological profiling. Molecular barcodes are short, unique stretches of nucleotides (DNA or RNA). Barcode reporters can be transcribed in response to receptor activation, serving as unique identifiers that quantitatively reflect cellular responses (Figure 3). Indeed, upon reporter activation, these barcodes are expressed and subsequently quantified by next-generation sequencing (NGS), enabling the simultaneous assessment of hundreds of receptors and pathways within a single well. This multiplexed strategy integrates transcriptional readouts with high-throughput sequencing, providing a scalable means to delineate hundreds to thousands of cellular activities. Such approaches have been implemented in massively parallelized reporter assays (MPRA) (Zheng and VanDusen, 2023) and multiplexed assays with optimized pathway reporters (Herholt et al., 2018; Chen et al., 2023; Wu et al., 2024; Trauernicht et al., 2024; Galinski et al., 2018; Zahm et al., 2024). Therefore, molecular barcodes can be used in genetically encoded multiplexed assays allowing the analysis of cellular disease mechanisms and pathological processes at once, screen drug candidates, and identify biomarkers more efficiently than traditional single-analyte assays.

### Multiplexed cell-based assays for GPCRs

Effective characterization of GPCRs requires robust and reproducible assays that can measure their activation and downstream signaling in living cells. Genetically encoded GPCR assays are typically divided into real-time, reporter gene, and cellular target-based categories, as well as into singleplex and multiplex formats (Wu et al., 2024). Multiplexed GPCR assays are advanced cell-based techniques designed to simultaneously generate multiple independent readouts from a single sample, offering a comprehensive view of many GPCR activities and various intracellular signaling cascades downstream of each receptor tested. Molecular barcoding as enabling technology is key to scale the multiplexing capacity of these assays.

**Figure 4:**
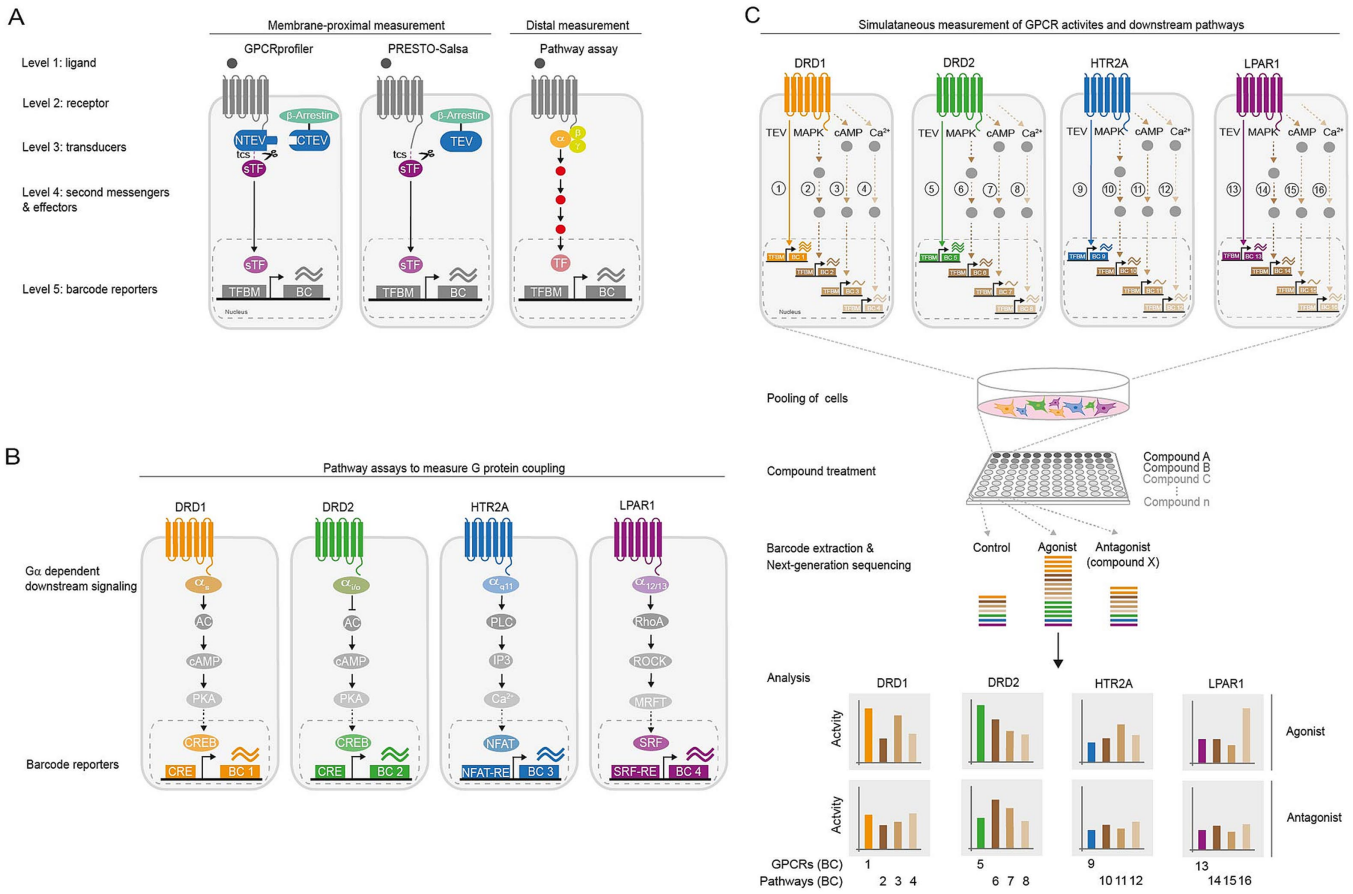
Barcoded receptor-proximal and pathway assays for the analysis of GPCR activities. **(A)** Split TEV (left) and full TEV (center) β-arrestin-2 recruitment assays, as well as functional pathway reporter assays (right), are used to measure the activity of GPCRs. GPCR activation and downstream signaling pathways can be measured at different hierarchical levels of cellular signaling, i.e., at the level of receptor (level 2), transducers (level 3), effectors and second messengers (level 4), and transcription factors (level 5). **(B)** Barcoded pathway assays to measure G protein coupling. Multiple GPCR pathway activities can be measured using responsive *cis*-regulatory elements that capture upstream activities of signaling pathways, such as CRE, NFAT-RE, or SRF-RE. Examples of Gαs, Gαi/o, Gαq, and Gα12/13 coupled GPCRs are DRD1, DRD2, HTR2A, and LPAR1. **(C)** Multiplexed assay workflow to simultaneously measure activities of multiple GPCRs and key downstream signaling pathways. Barcoded reporters are used for receptor-proximal activity assays (split TEV or full TEV) and distal pathway assays (MAPK, cAMP, and Ca^2+^ signaling pathways). Cells expressing a given receptor and assay components are pooled and plated into 96-well plates, where the compounds are tested. Following the cell lysis, barcodes (BCs) are extracted and sequenced with next-generation sequencing (NGS). Sequencing results are analysed and visualized as a target and pathway profile activity plot. The bar plots serve as illustrative examples of the target profiles. CRE, cAMP-responsive element; NFAT-RE, calcium sensor nuclear factor of activated T cells response element; SRF-RE, serum response factor response element; DRD1/2, dopamine receptor 1/2; HTR2A, serotonin receptor 2A; LPAR1, lysophosphatidic acid receptor 1; MAPK, mitogen-activated protein kinase; cyclic cAMP, adenosine 3′,5′-monophosphate; Ca^2+^, calcium; TFBM, transcription factor binding motif; TF, transcription factor; sTF, synthetic transcription factor; AC, adenylate cyclase; PKA, protein kinase A; PLC, phospholipase C; IP3, inositol 1,4,5-trisphosphate; ROCK, Rho-associated coiled-coil containing kinase; MRTF, myocardin-related transcription factor.

Barcoded GPCR assays can be broadly distinguished by whether they measure receptor-proximal events at the membrane or downstream signaling outputs in the nucleus (Figure 4A). Barcoded GPCR assays that track receptor activity at the membrane use an activity dependent recruitment of a protein, typically a truncated version of β-arrestin-2 that directly interacts with activated, desensitized receptors, causing the release of an artificial co-transcriptional activator as reporter molecule that transcriptionally activates a barcoded reporter from a synthetic promotor. Examples of barcoded membrane GPCR assays are the GPCRprofiler assay, which uses a split TEV GPCR β-arrestin-2 recruitment strategy (Figure 4A, left panel) in transiently transfected cells for monitoring the activity of 19 different GPCRs (Galinski et al., 2018), and the PRESTO-Salsa platform, a full TEV protease-based β-arrestin-2 recruitment assay (Figure 4A, middle panel) for interrogation of the druggable human GPCRome, built upon PRESTO-Tango (Kroeze et al., 2015), which enables the simultaneous assessment of more than 300 receptors within a single well of a 96-well plate (Chen et al., 2023).

By contrast, barcoded GPCR pathway assays measure receptor activation at the distal end of signaling cascades in the nucleus using reporter gene assays (Figure 4A, right panel). These assays allow the simultaneous measurement of various cellular pathways downstream of GPCR activation, such as cAMP-responsive element (CRE), MAP kinase (MAPK) signaling, or nuclear factor of activated T cells (NFAT-RE) pathways (Popović et al., 2024a) (Figure 4B). Recently, the systemic optimisation of 325 transcription factor binding motifs (TFBMs) as short synthetic pathway sensors was conducted to profile the activity of multiple pathways downstream of GPCRs (Zahm et al., 2024). Therefore, this tool allows for the precise and sensitive quantification of transcriptional responses to various cellular stimuli, offering significant utility for drug discovery, synthetic biology, and the elucidation of GPCR-mediated cellular signaling. However, Gαi/o-coupled GPCR activities could not be measured using this method in HEK293 cells, arguing that receptor-proximal activity measurements, e.g., based on full-TEV (Chen et al., 2023) and split-TEV (Galinski et al., 2018) β-arrestin recruitment assays, can complement these assays.

By contrast to barcoded GPCR assays described above, which measure either direct target activities or cumulated downstream activities, a recent strategy employs the co-expression of individual receptors, constructs for split TEV recruitment assays, and functional pathway reporters carrying distinct molecular barcodes. This enables the pooled analysis of multiple GPCR activities, capturing both direct membrane-proximal signaling via the split TEV β-arrestin-2 recruitment assay and key downstream pathway activities in a single well assay (Popović et al., 2024a) (Figure 4C). By allowing systematic evaluation of both on-target and off-target as well as on-pathway and off-pathway activities across multiple receptors and their downstream signaling pathways, multiplexed assays provide a robust approach to dissect GPCR polypharmacology and pathway crosstalk, thereby informing drug development (Wu et al., 2024).

Pathway effects can be measured more precisely for a given disease by conducting profiling assays in cell types that reflect the relevant physiological context, such as neurons for neurological or psychiatric disorders. However, iPSC-derived neurons remain technically challenging due to higher standards in ensuring neuronal maturity and functionality as well as maintaining consistency and reproducibility (Mateos-Aparicio et al., 2020).

Integrating barcoded assay technologies may help overcome these limitations by enabling multiplexed and high-resolution molecular readouts that offer new insights into neuronal pathway biology. In this regard, the barcoded pathwayProfiler assay that employs both synthetic and clustered TFBMs as well as endogenous promoters as pathway reporters, has been optimized for measuring stimuli in primary mouse neurons (Herholt et al., 2018) and was used to assess dysregulations of signaling caused by the autism and schizophrenia risk gene TAOK2 (Ma et al., 2025). *Taok2*-deficient neurons exhibited reduced calcium and MAPK–ERK signaling in response to AMPA, brain-derived neurotrophic factor (BDNF), which is the ligand for the RTK TrkB, and bicuculine, a GABA-A receptor antagonist. These altered signaling properties result in impaired synaptic plasticity, reduced dendritic complexity, and an anxiety phenotype in mice. Together, these findings support the use of barcoded pathway profiling assays as early-stage screening tools for identifying and characterizing druggable targets. Pathway sensors identified in these studies could be directly incorporated into barcoded profiling assays to monitor GPCR-dependent regulation of disease-relevant genes.

All barcoded GPCR profiling assays reported to date have been conducted using transient transfection of plasmids encoding GPCRs and reporter components, except for of the β-arrestin-2-full-TEV element, which was stably integrated in HEK293 cells for the PRESTO-Salsa platform (Chen et al., 2023). Despite this flexibility, also for normalization strategies to identify cell-type dependent and compound-dependent effects (Popović et al., 2024b), transient transfection has several limitations that compromise its effectiveness, especially in long-term or high-throughput assays. The effect on target gene expression can only be sustained for a limited period, and the results are not reproducible due to variability (Chong et al., 2021). In contrast, the stable integration of genetic elements, either the GPCR, reporter components, or both, provides a solution to these limitations by improving assay consistency, sensitivity, and cost-effectiveness.

When selecting the most appropriate barcoded GPCR assay platform, the purpose of the assay needs to be considered. For example, multi-target drugs may be profiled for agonist, antagonist, or inverse agonist features. Published barcoded GPCR assay systems are well-suited for agonist and antagonist profiling. However, for inverse agonists, a higher constitutive activity of a given GPCR may be required. This could be achieved by integrating the cumate-inducible expression system into the barcoded assays (Zeghal et al., 2023). Using barcoded assay platforms, GPCR activity can be monitored receptor-proximally using full-TEV and split-TEV approaches or distally using functional pathway reporters (Galinski et al., 2018; Chen et al., 2023; Popović et al., 2024b; Zahm et al., 2024). While TEV-based assays measure GPCR activity using overexpressed, synthetic constructs, pathway reporters capture endogenous signaling. Furthermore, it may be critical to monitor compound effects on signaling not only in heterologous cell lines, but also in neurons to increase translatability. To translate the advantages and limitations of barcoded GPCR assays into actionable guidance, we developed a decision matrix for assay selection (see Table 1 for details). We anticipate that future assay development, particularly for the type of delivery and adjusted expression of the genetically engineered assay elements, will promote robust and reproducible GPCR pharmacology. Such improvements will facilitate the screening of large compound libraries by capturing desired pathway-specific activities while reducing the influence of unintended signaling events within a single measurement.

**Table 1:** Comparison of barcoded GPCR assay platforms.

Parameters	Barcoded receptor assays		Barcoded pathway assays
Method	Full-TEV (Tango)	Split-TEV	Functional reporter assay
Ligands to study	Agonists, antagonists, inverse agonists (the latter only with high basal activity), partial agonists		
Receptor status	Chimeric constructs		Native or overexpressed
Readout level	Proximal (β-arrestin-2 recruitment), direct reporter assay		Distal (transcription factor activity)
Assay type	Transfection, β-arrestin-2-TEV stable line	Transfection	Transfection, viral infection, stable lines
Cell type	HEK293	HEK293, PC12	HEK293, PC12, neurons
Multiplexing	100 s of GPCRs/experiment		Multiple pathways/experiment
Publication status	Published (Chen et al., 2023)	Published (Galinski et al., 2018; Popović et al., 2024b)	Published (Popović et al., 2024b; Zahm et al., 2024; Ma et al., 2025)

	Advantages		
Signal amplification	Double amplification: proteolytic cleavage + transcriptional		Transcriptional amplification
Specificity	Receptor-specific; discriminates from endogenous GPCR activity		Preserves endogenous signaling context
Signal-to-noise ratio	High	High; lower background than full-TEV due to reconstitution-dependent protease activation	High; very low background with robust and tight reporters
GPCR coverage	Described for >300 GPCRs	Compatible with broad GPCR range; PC12 extends to neuronal context	Applicable to any GPCR coupled to monitored pathway
Pathway coverage	Focused β-arrestin readout		Multiple pathways simultaneously
Combinability	Combinable with barcoded pathway assays		
Throughput	Pooled screening compatible; high-throughput; stable lines improve robustness		

	Limitations		
Receptor modifications	Chimeric constructs required (GPCR–TEV, truncated β-arr2–TEV)	Chimeric constructs required (GPCR, truncated β-arr2–TEV split fragments)	Pathway reporters require cell engineering; receptor can be native
Endogenous interference	Endogenous β-arr2 competes; may reduce dynamic range		Less pronounced; pathway crosstalk possible
Pathway bias	β-arrestin only; no G-protein or other signaling arms		Dependent on reporter panel; non-canonical pathways may be missed
Leakiness	Higher leakiness than split TEV; constitutively active full TEV	Reduced vs. full TEV; residual spontaneous reconstitution possible	Dependent on promoter and cell line
Selectivity, efficacy, potency	Chimeric receptors may alter pharmacology profiling		Native receptor context better suits profiling
Temporal resolution	Slow (transcriptional readout); not suitable for kinetic studies		
TEV cytotoxicity	Full-TEV may be cytotoxic at high expression	Reduced risk; split-TEV fragments individually inactive	None
Assay development	Well-established	Well-established; requires validation of reconstituted TEV protease activity	Stable reporter lines require upfront development
Inverse agonists	Limited profiling, as a high constitutive (basal) activity is required		Better detection
Weak partial agonists	Detection threshold limited by protease activation requirement	Reconstitution requirement may reduce sensitivity	Transcriptional amplification aids detection; slow kinetics limit time-resolved measurements

### GPCRs as relevant targets for the safety profiling of drugs

The success rate for drug candidates that enter clinical development and ultimately reach approval is generally considered at around 10%, while about 90% fail, resulting in substantial financial losses (Sun et al., 2022). A recent benchmark study reported a slightly higher approval rate of 14%, yet attrition during clinical development remains high (Schuhmacher et al., 2025). Although drug discovery is guided by rigorous evaluation throughout the entire process, approved and marketed drugs are still occasionally withdrawn for safety reasons, albeit in rates in the low single-digit percent range, leading to additional financial costs (Rawson, 2016). Most failures are attributed to safety-related causes such as hepatotoxicity, abuse potential, or insufficient efficacy, and these effects are often linked to compound promiscuity and off-target activities (Onakpoya et al., 2016).

**Figure 5:**
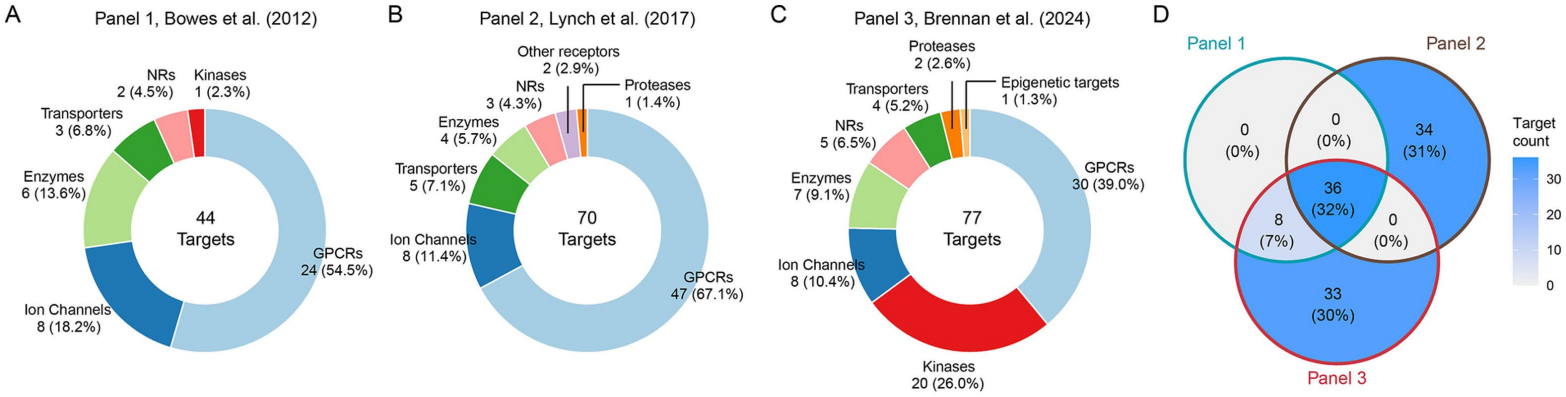
Development of drug target panels for safety profiling from 2012 to today **(A–C)**. Donut charts showing relevant safety profiling panels. The first safety profiling panel was introduced by Bowes et al. in 2012 (Bowes et al., 2012), encompassing 44 targets **(A)**. In 2017, Lynch et al. (2017) proposed to use a refined version with 70 targets **(B)**. The most recent safety profiling panel was introduced by Brennan et al. in 2024 (Brennan et al., 2024) with 77 targets **(C)**. **(D)** Venn diagram showing the target overlap for the three panels. Panels include GPCRs, ion channels, transporters, enzymes, nuclear receptors, and kinases. See Supplementary Table S2 for a detailed description of the drug targets and their classes for each safety profiling panel.

To mitigate these risks, safety pharmacology profiling emerged as an early-stage strategy to evaluate the off-target interactions of drug candidates before preclinical or clinical testing. In 2012, four major pharmaceutical companies, i.e., AstraZeneca, GlaxoSmithKline, Novartis, and Pfizer, defined the first safety-screening panel that consisted of 44 drug targets (Bowes et al., 2012) (Figure 5A). Notably, 24 of these (~ 55%) were GPCRs, emphasizing the central importance of this receptor class in both drug discovery and safety evaluation. For instance, activating HRH1 may trigger hypotension or allergic responses, whereas blocking it can lead to sedation and weight gain (Walsh, 2002). Likewise, DRD1 receptor agonists, in particular older generations, are associated with adverse effects, such as receptor desensitization, seizures, and dyskinesias, making this receptor a key target to be evaluated in drug discovery programs for psychiatric disorders and PD (Jones-Tabah et al., 2022). A second safety profiling panel was introduced in 2017, now covering 70 targets (Lynch et al., 2017) (Figure 5B). Beyond GPCRs, early safety panels also included ion channels, enzymes, nuclear receptors, transporters, and a kinase.

A recent cross-industry analysis confirmed that these receptor families remain central to safety pharmacology (Brennan et al., 2024). The current and updated secondary pharmacology panel now includes 77 targets, with 30 additional GPCRs and several kinase and ion-channel receptors (Figure 5C). Brennan et al. (2024) suggested conducting an initial small-scale test of the panel. If no off-target effects were identified, further testing of the remaining panels was necessary. Panels that failed the initial test were discarded, or the drug was adjusted accordingly.

There is a significant overlap among the three panels, particularly to the first panel, showing the overall consistency of this approach (Figure 5D; Supplementary Table S2). Since the introduction of standardized safety profiling, the incidence of off-target–related adverse effects in marketed drugs from 2010 to 2019 has decreased by approximately 25–30%, showing that a broader use of standardized selectivity profiling has promoted the safety of newer small-molecule drugs. This expanded framework, now widely used across the pharmaceutical industry, refines early safety assessment while keeping GPCRs at its core.

### Barcoded cell-based assays are a suitable platform to assess the activity of multiple targets, also beyond GPCRs

The safetyProfiler platform expands the versatility of barcoded pathway assays by enabling the parallel profiling of multiple GPCRs, receptor tyrosine kinases (RTKs), nuclear receptors and proteolytic events, as well as key signaling pathways within a single experiment (Popović et al., 2024c). While the direct activity of GPCRs was monitored using the split TEV β-arrestin-2 recruitment assay, activities of RTKs were measured with a split TEV GRB2 recruitment assay. Activities of nuclear receptors were monitored using selective TFBMs, and the activity of a gamma secretase activity was measured through the selective activation of a synthetic transcription factor. This approach provided a comprehensive view of compound activity across diverse receptor classes, thereby uncovering complex polypharmacological interactions and offering a powerful framework for systematic safety and efficacy assessment. To note here, the choice of cell lines is a critical factor in such drug profiling assays, particularly for platforms like the safetyProfiler assay. The selection process is guided by several key considerations, including the expression of receptors and their endogenous binding partners required for downstream signaling, general signaling competence, and physiological relevance, to ensure robust, reproducible, and translatable results. Indeed, different cell lines exhibit varying performance levels for specific drug targets (Hodos et al., 2018). While some assays perform better in specific non-human cell lines (like rat PC12), there’s a strong emphasis on using human cells, particularly HEK293, whenever applicable, to ensure greater translatability to human biology (Popović et al., 2024a).

As GPCRs constitute one of the most pharmacologically diverse receptor families, safety profiling not only serves to identify potential off-target risks but also uncovers opportunities for secondary pharmacology. Systematic profiling of GPCR activities can reveal previously unrecognized beneficial interactions, guiding drug repurposing and the discovery of known and novel therapeutics for other indications. For example, a recent study identified several FDA-approved GPCR-targeting drugs with anticancer potential through high-throughput screening in breast cancer models: Notably, the β-adrenergic receptor antagonist nebivolol, originally developed for cardiovascular disorders, showed significant inhibition of tumor cell proliferation, migration, and invasion (Abdulkareem et al., 2022). Another classic example is clemastine, an HRH1 antihistamine that was later found to exert off-target antagonism of the muscarinic acetylcholine receptor CHRM1, enabling its repurposing as a remyelinating therapy for MS (Cunniffe and Coles, 2021). Such repurposing driven by early safety data exemplifies how modern profiling strategies can transform safety evaluation from a purely preventive measure into a creative engine for therapeutic innovation.

## Conclusion

Multiplexed cell-based assays provide a large capacity for the simultaneous profiling of multiple targets and pathways. Therefore, these powerful platforms are expected to accelerate GPCR drug discovery by enabling a more complete assessment of compound effects and overcome the limitations of singleplex assays, which provide only one data point per experimental condition. By enabling target and pathway-level screening technologies that can distinguish desired therapeutic effects from adverse off-target and cross-pathway signaling effects, multiplexed assays in early discovery pipelines will help to accelerate the identification of promising GPCR-targeting candidates while allowing cost-effective screening of large compound libraries (Wu et al., 2024; Heesen et al., 2026). In addition, opportunities for secondary pharmacology of compounds, e.g., by guiding drug repurposing and by identifying an additional therapeutic use, may be substantially increased, further unlocking the full potential of these broad profiling assays for drug development. Importantly, as GPCRs represent a major therapeutic opportunity across CNS diseases and disorders, the integration of multiplexed approaches has the potential to drive the discovery of more precise and effective treatments by revealing complex signaling dynamics that underlie neurological dysfunction.

## Supplementary material

SUPPLEMENTARY TABLE S1 GPCRs implicated in neurodegenerative, neurodevelopmental, and psychiatric disorders. GPCR mean association score and disease associations were retrieved from GPCRdb.

SUPPLEMENTARY TABLE S2 Targets of safety profiling panels.

## References

[ref1] Abdulkareem N. M. Bhat R. Powell R. T. Chikermane S. Yande S. Trinh L. . (2022). Screening of GPCR drugs for repurposing in breast cancer. Front. Pharmacol. 13:1049640. doi: 10.3389/fphar.2022.1049640, 36561339 PMC9763283

[ref2] Ackmann J. Brüge A. Gotina L. Lim S. Jahreis K. Vollbrecht A.-L. . (2024). Structural determinants for activation of the tau kinase CDK5 by the serotonin receptor 5-HT7R. Cell Commun. Signal 22:233. doi: 10.1186/s12964-024-01612-y, 38641599 PMC11031989

[ref3] Aires I. D. Boia R. Rodrigues-Neves A. C. Madeira M. H. Marques C. Ambrósio A. F. . (2019). Blockade of microglial adenosine A2A receptor suppresses elevated pressure-induced inflammation, oxidative stress, and cell death in retinal cells. Glia 67, 896–914. doi: 10.1002/glia.23579, 30667095 PMC6590475

[ref4] Albert P. R. (2015). Why is depression more prevalent in women? J. Psychiatry Neurosci. JPN 40, 219–221. doi: 10.1503/jpn.150205, 26107348 PMC4478054

[ref5] Alessandri M. Osorio-Forero A. Lüthi A. Chatton J.-Y. (2024). The lactate receptor HCAR1: A key modulator of epileptic seizure activity. iScience 27:109679. doi: 10.1016/j.isci.2024.109679, 38655197 PMC11035371

[ref6] Annamneedi A. Gora C. Dudas A. Leray X. Bozon V. Crépieux P. . (2025). Towards the convergent therapeutic potential of G protein-coupled receptors in autism spectrum disorders. Br. J. Pharmacol. 182, 3044–3067. doi: 10.1111/bph.16216, 37574491

[ref7] Ao C. Li C. Chen J. Tan J. Zeng L. (2022). The role of Cdk5 in neurological disorders. Front. Cell. Neurosci. 16:951202. doi: 10.3389/fncel.2022.951202, 35966199 PMC9368323

[ref8] Aosaki T. Miura M. Suzuki T. Nishimura K. Masuda M. (2010). Acetylcholine-dopamine balance hypothesis in the striatum: an update. Geriatr Gerontol Int 10 Suppl 1, S148–S157. doi: 10.1111/j.1447-0594.2010.00588.x, 20590830

[ref9] Aronica E. Catania M. V. Geurts J. Yankaya B. Troost D. (2001). Immunohistochemical localization of group I and II metabotropic glutamate receptors in control and amyotrophic lateral sclerosis human spinal cord: upregulation in reactive astrocytes. Neuroscience 105, 509–520. doi: 10.1016/S0306-4522(01)00181-6, 11672616

[ref10] Arroll B. Macgillivray S. Ogston S. Reid I. Sullivan F. Williams B. . (2005). Efficacy and tolerability of tricyclic antidepressants and SSRIs compared with placebo for treatment of depression in primary care: A Meta-analysis. Ann. Fam. Med. 3, 449–456. doi: 10.1370/afm.349, 16189062 PMC1466912

[ref11] Azam S. Haque M. E. Jakaria M. Jo S.-H. Kim I.-S. Choi D.-K. (2020). G-protein-coupled receptors in CNS: A potential therapeutic target for intervention in neurodegenerative disorders and associated cognitive deficits. Cells 9:506. doi: 10.3390/cells9020506, 32102186 PMC7072884

[ref12] Badria F. A. De Filippis B. El-Magd M. A. Elbadawi M. M. Hamdi A. Elgazar A. A. (2025). Editorial: multi-target drug discovery and design for complex health disorders. Front. Pharmacol. 16:1633600. doi: 10.3389/fphar.2025.1633600, 40584615 PMC12202662

[ref13] Barbeau A. (1962). The pathogenesis of Parkinson’s disease: a new hypothesis. Can. Med. Assoc. J. 87, 802–807. 13966498 PMC1849683

[ref14] Battaglia G. Bruno V. (2018). Metabotropic glutamate receptor involvement in the pathophysiology of amyotrophic lateral sclerosis: new potential drug targets for therapeutic applications. Curr. Opin. Pharmacol. 38, 65–71. doi: 10.1016/j.coph.2018.02.007, 29529498

[ref15] Beversdorf D. Q. Nordgren R. E. Bonab A. A. Fischman A. J. Weise S. B. Dougherty D. D. . (2012). 5-HT2 receptor distribution shown by [18F] setoperone PET in high-functioning autistic adults. J. Neuropsychiatry Clin. Neurosci. 24, 191–197. doi: 10.1176/appi.neuropsych.11080202, 22772667

[ref16] Blázquez C. Chiarlone A. Sagredo O. Aguado T. Pazos M. R. Resel E. . (2011). Loss of striatal type 1 cannabinoid receptors is a key pathogenic factor in Huntington’s disease. Brain 134, 119–136. doi: 10.1093/brain/awq278, 20929960

[ref17] Bloem B. R. Okun M. S. Klein C. (2021). Parkinson’s disease. Lancet (Lond. Engl.). 397, 2284–2303. doi: 10.1016/S0140-6736(21)00218-X 33848468

[ref18] Boczek T. Mackiewicz J. Sobolczyk M. Wawrzyniak J. Lisek M. Ferenc B. . (2021). The role of G protein-coupled receptors (GPCRs) and calcium signaling in schizophrenia. Focus on GPCRs activated by neurotransmitters and chemokines. Cells 10:1228. doi: 10.3390/cells10051228, 34067760 PMC8155952

[ref19] Bolinger A. A. Frazier A. La J.-H. Allen J. A. Zhou J. (2023). Orphan G protein-coupled receptor GPR37 as an emerging therapeutic target. ACS Chem. Neurosci. 14, 3318–3334. doi: 10.1021/acschemneuro.3c00479, 37676000 PMC11144446

[ref20] Bowes J. Brown A. J. Hamon J. Jarolimek W. Sridhar A. Waldron G. . (2012). Reducing safety-related drug attrition: the use of in vitro pharmacological profiling. Nat. Rev. Drug Discov. 11, 909–922. doi: 10.1038/nrd3845, 23197038

[ref21] Brandenburg C. Soghomonian J.-J. Zhang K. Sulkaj I. Randolph B. Kachadoorian M. . (2020). Increased dopamine type 2 gene expression in the dorsal striatum in individuals with autism Spectrum disorder suggests alterations in indirect pathway signaling and circuitry. Front. Cell. Neurosci. 14:577858. doi: 10.3389/fncel.2020.577858, 33240045 PMC7681004

[ref22] Brennan R. J. Jenkinson S. Brown A. Delaunois A. Dumotier B. Pannirselvam M. . (2024). The state of the art in secondary pharmacology and its impact on the safety of new medicines. Nat. Rev. Drug Discov. 23, 525–545. doi: 10.1038/s41573-024-00942-3 38773351

[ref23] Brisch R. Saniotis A. Wolf R. Bielau H. Bernstein H.-G. Steiner J. . (2014). The role of dopamine in schizophrenia from a neurobiological and evolutionary perspective: old fashioned, but still in vogue. Front. Psych. 5:47. doi: 10.3389/fpsyt.2014.00047, 24904434 PMC4032934

[ref24] Brust T. F. Conley J. M. Watts V. J. (2015). Gα(i/o)-coupled receptor-mediated sensitization of adenylyl cyclase: 40 years later. Eur. J. Pharmacol. 763, 223–232. doi: 10.1016/j.ejphar.2015.05.014, 25981304 PMC4584185

[ref25] Cao W. Luttrell L. M. Medvedev A. V. Pierce K. L. Daniel K. W. Dixon T. M. . (2000). Direct binding of activated c-Src to the β3-adrenergic receptor is required for MAP kinase activation *. J. Biol. Chem. 275, 38131–38134. doi: 10.1074/jbc.C000592200, 11013230

[ref26] Caprariello A. V. Adams D. J. (2022). The landscape of targets and lead molecules for remyelination. Nat. Chem. Biol. 18, 925–933. doi: 10.1038/s41589-022-01115-2, 35995862 PMC9773298

[ref27] Cataldo I. Azhari A. Esposito G. (2018). A review of oxytocin and arginine-vasopressin receptors and their modulation of autism Spectrum disorder. Front. Mol. Neurosci. 11:27. doi: 10.3389/fnmol.2018.00027, 29487501 PMC5816822

[ref28] Caulfield M. E. Manfredsson F. P. Steece-Collier K. (2023). “The role of striatal Cav1.3 calcium channels in therapeutics for Parkinson’s disease,” in Voltage-Gated Ca2+ Channels: Pharmacology, Modulation and their Role in Human Disease, ed. Striessnig J. (Cham: Springer International Publishing), 107–137. 10.1007/164_2022_629 36592226

[ref29] Celorrio M. Rojo-Bustamante E. Fernández-Suárez D. Sáez E. de Estella-Hermoso Mendoza A. Müller C. E. . (2017). GPR55: a therapeutic target for Parkinson’s disease? Neuropharmacology 125, 319–332. doi: 10.1016/j.neuropharm.2017.08.017, 28807673

[ref30] Chakraborty P. Ibáñez de Opakua A. Purslow J. A. Fromm S. A. Chatterjee D. Zachrdla M. . (2024). GSK3β phosphorylation catalyzes the aggregation of tau into Alzheimer’s disease-like filaments. Proc. Natl. Acad. Sci. USA 121:e2414176121. doi: 10.1073/pnas.2414176121, 39693350 PMC11670061

[ref31] Chen J.-F. Cunha R. A. (2020). The belated US FDA approval of the adenosine A2A receptor antagonist istradefylline for treatment of Parkinson’s disease. Purinergic Signal 16, 167–174. doi: 10.1007/s11302-020-09694-2, 32236790 PMC7367999

[ref32] Chen Y. Peng Y. Che P. Gannon M. Liu Y. Li L. . (2014). Α(2A) adrenergic receptor promotes amyloidogenesis through disrupting APP-SorLA interaction. Proc. Natl. Acad. Sci. USA 111, 17296–17301. doi: 10.1073/pnas.1409513111, 25404298 PMC4260556

[ref33] Chen H. Rosen C. E. González-Hernández J. A. Song D. Potempa J. Ring A. M. . (2023). Highly multiplexed bioactivity screening reveals human and microbiota metabolome-GPCRome interactions. Cell 186, 3095–3110.e19. doi: 10.1016/j.cell.2023.05.024, 37321219 PMC10330796

[ref34] Chen Q. Tesmer J. J. G. (2022). G protein–coupled receptor interactions with arrestins and GPCR kinases: the unresolved issue of signal bias. J. Biol. Chem. 298:102279. doi: 10.1016/j.jbc.2022.102279, 35863432 PMC9418498

[ref35] Chong Z. X. Yeap S. K. Ho W. Y. (2021). Transfection types, methods and strategies: a technical review. PeerJ 9:e11165. doi: 10.7717/peerj.11165, 33976969 PMC8067914

[ref36] Chundi A. Pham U. Darbha S. Rajagopal S. (2025). Biased agonists of the type 1 angiotensin II receptor promote distinct subcellular β-Arrestin conformations. Biochemistry 64, 4206–4216. doi: 10.1021/acs.biochem.4c00884, 40974303 PMC13267248

[ref37] Comerford I. McColl S. R. (2024). Atypical chemokine receptors in the immune system. Nat. Rev. Immunol. 24, 753–769. doi: 10.1038/s41577-024-01025-5, 38714818

[ref38] Corti S. Locatelli F. Donadoni C. Guglieri M. Papadimitriou D. Strazzer S. . (2004). Wild-type bone marrow cells ameliorate the phenotype of SOD1-G93A ALS mice and contribute to CNS, heart and skeletal muscle tissues. Brain J. Neurol. 127, 2518–2532. doi: 10.1093/brain/awh273, 15469951

[ref39] Cui L. Li S. Wang S. Wu X. Liu Y. Yu W. . (2024). Major depressive disorder: hypothesis, mechanism, prevention and treatment. Signal Transduct. Target. Ther. 9:30. doi: 10.1038/s41392-024-01738-y, 38331979 PMC10853571

[ref40] Cunniffe N. Coles A. (2021). Promoting remyelination in multiple sclerosis. J. Neurol. 268, 30–44. doi: 10.1007/s00415-019-09421-x, 31190170 PMC7815564

[ref41] De Deurwaerdère P. Ponimaskin E. Chagraoui A. Di Giovanni G. (2022). Editorial: new GPCR targets and modulators to treat CNS disorders. Front. Mol. Neurosci. 15:1104336. doi: 10.3389/fnmol.2022.1104336, 36561894 PMC9763925

[ref42] Debanne D. Mylonaki K. Musella M. L. Russier M. (2024). Voltage-gated ion channels in epilepsies: circuit dysfunctions and treatments. Trends Pharmacol. Sci. 45, 1018–1032. doi: 10.1016/j.tips.2024.09.004, 39406591

[ref43] Desale S. E. Chidambaram H. Chinnathambi S. (2021). G-protein coupled receptor, PI3K and rho signaling pathways regulate the cascades of tau and amyloid-β in Alzheimer’s disease. Mol. Biomed. 2:17. doi: 10.1186/s43556-021-00036-1, 35006431 PMC8607389

[ref44] Devinsky O. Vezzani A. O’Brien T. J. Jette N. Scheffer I. E. de Curtis M. . (2018). Epilepsy. *Nat. Rev. Dis. Primer* 4:18024. doi: 10.1038/nrdp.2018.24 29722352

[ref45] Dhapola R. Sharma P. Kumari S. Vellingiri B. Medhi B. HariKrishnaReddy D. (2025). Exploring retinal neurodegeneration in Alzheimer’s disease: A molecular and cellular perspective. Neurotox. Res. 43:22. doi: 10.1007/s12640-025-00744-4, 40216597

[ref46] Di Menna L. Alborghetti M. De Bartolo M. I. Borro M. Gentile G. Zinni M. . (2025). Preclinical and clinical study on type 3 metabotropic glutamate receptors in Parkinson’s disease. Npj Park. Dis. 11:9. doi: 10.1038/s41531-024-00860-6, 39755677 PMC11700162

[ref47] Dobson R. Giovannoni G. (2019). Multiple sclerosis – a review. Eur. J. Neurol. 26, 27–40. doi: 10.1111/ene.13819, 30300457

[ref48] Doria J. G. de Souza J. M. Andrade J. N. Rodrigues H. A. Guimaraes I. M. Carvalho T. G. . (2015). The mGluR5 positive allosteric modulator, CDPPB, ameliorates pathology and phenotypic signs of a mouse model of Huntington’s disease. Neurobiol. Dis. 73, 163–173. doi: 10.1016/j.nbd.2014.08.021, 25160573

[ref49] Doria J. G. de Souza J. M. Silva F. R. Olmo I. G. Carvalho T. G. Alves-Silva J. . (2018). The mGluR5 positive allosteric modulator VU0409551 improves synaptic plasticity and memory of a mouse model of Huntington’s disease. J. Neurochem. 147, 222–239. doi: 10.1111/jnc.14555 30028018 PMC6317718

[ref50] Doze V. A. Handel E. M. Jensen K. A. Darsie B. Luger E. J. Haselton J. R. . (2009). α1A- and α1B-adrenergic receptors differentially modulate antidepressant-like behavior in the mouse. Brain Res. 1285, 148–157. doi: 10.1016/j.brainres.2009.06.035, 19540213 PMC2720445

[ref51] Ejiohuo O. Bilska K. Narożna B. Skibińska M. Kapelski P. Dmitrzak-Węglarz M. . (2025). The implication of *ADRA2A* and *AVPRIB* gene variants in the aetiology of stress-related bipolar disorder. J. Affect. Disord. 368, 249–257. doi: 10.1016/j.jad.2024.09.072 39278467

[ref52] Falkai P. Rossner M. J. Schulze T. G. Hasan A. Brzózka M. M. Malchow B. . (2015). Kraepelin revisited: schizophrenia from degeneration to failed regeneration. Mol. Psychiatry 20, 671–676. doi: 10.1038/mp.2015.35, 25824303

[ref53] Felder C. C. Goldsmith P. J. Jackson K. Sanger H. E. Evans D. A. Mogg A. J. . (2018). Current status of muscarinic M1 and M4 receptors as drug targets for neurodegenerative diseases. Neuropharmacology 136, 449–458. doi: 10.1016/j.neuropharm.2018.01.028, 29374561

[ref54] Fouillen A. Bous J. Granier S. Mouillac B. Sounier R. (2023). Bringing GPCR structural biology to medical applications: insights from both V2 vasopressin and mu-opioid receptors. Membranes 13:606. doi: 10.3390/membranes13060606, 37367810 PMC10303988

[ref55] Fredriksson R. Lagerström M. C. Lundin L.-G. Schiöth H. B. (2003). The G-protein-coupled receptors in the human genome form five Main families. Phylogenetic analysis, Paralogon groups, and fingerprints. Mol. Pharmacol. 63, 1256–1272. doi: 10.1124/mol.63.6.1256, 12761335

[ref56] Fu J. M. Satterstrom F. K. Peng M. Brand H. Collins R. L. Dong S. . (2022). Rare coding variation provides insight into the genetic architecture and phenotypic context of autism. Nat. Genet. 54, 1320–1331. doi: 10.1038/s41588-022-01104-0, 35982160 PMC9653013

[ref57] Gainetdinov R. R. Bohn L. M. Walker J. K. Laporte S. A. Macrae A. D. Caron M. G. . (1999). Muscarinic supersensitivity and impaired receptor desensitization in G protein-coupled receptor kinase 5-deficient mice. Neuron 24, 1029–1036. doi: 10.1016/s0896-6273(00)81048-x 10624964

[ref58] Galinski S. Wichert S. P. Rossner M. J. Wehr M. C. (2018). Multiplexed profiling of GPCR activities by combining split TEV assays and EXT-based barcoded readouts. Sci. Rep. 8:8137. doi: 10.1038/s41598-018-26401-9, 29802268 PMC5970223

[ref59] Gao F. Yang S. Wang J. Zhu G. (2022). cAMP-PKA cascade: an outdated topic for depression? Biomed. Pharmacother. 150:113030. doi: 10.1016/j.biopha.2022.113030, 35486973

[ref60] Gassmann M. Stawarski M. Antonarakis S. E. Bettler B. (2025). Genetic implication of GABAB receptors in the etiology of neurological and psychiatric disorders. Front. Pharmacol. 16:1634028. doi: 10.3389/fphar.2025.1634128, 40756990 PMC12314290

[ref61] GBD 2021 Nervous System Disorders Collaborators (2024). Global, regional, and national burden of disorders affecting the nervous system, 1990-2021: a systematic analysis for the global burden of disease study 2021. Lancet Neurol. 23, 344–381. doi: 10.1016/S1474-4422(24)00038-3, 38493795 PMC10949203

[ref62] Gerfen C. R. Surmeier D. J. (2011). Modulation of striatal projection systems by dopamine. Annu. Rev. Neurosci. 34, 441–466. doi: 10.1146/annurev-neuro-061010-113641, 21469956 PMC3487690

[ref63] Gibson J. S. Claassen D. O. (2021). State-of-the-art pharmacological approaches to reduce chorea in Huntington’s disease. Expert. Opin. Pharmacother. 22, 1015–1024. doi: 10.1080/14656566.2021.1876666, 33550875 PMC8222076

[ref64] Gillman P. K. (2007). Tricyclic antidepressant pharmacology and therapeutic drug interactions updated. Br. J. Pharmacol. 151, 737–748. doi: 10.1038/sj.bjp.0707253, 17471183 PMC2014120

[ref65] Grove J. Ripke S. Als T. D. Mattheisen M. Walters R. K. Won H. . (2019). Identification of common genetic risk variants for autism spectrum disorder. Nat. Genet. 51, 431–444. doi: 10.1038/s41588-019-0344-8 30804558 PMC6454898

[ref66] Gurevich V. V. Gurevich E. V. (2017). Molecular mechanisms of GPCR signaling: A structural perspective. Int. J. Mol. Sci. 18:2519. doi: 10.3390/ijms18122519, 29186792 PMC5751122

[ref67] Haas L. T. Strittmatter S. M. (2016). Oligomers of amyloid β prevent physiological activation of the cellular prion protein-metabotropic glutamate receptor 5 complex by glutamate in Alzheimer disease. J. Biol. Chem. 291, 17112–17121. doi: 10.1074/jbc.M116.720664, 27325698 PMC5016115

[ref68] Heesen S. H. Chang M.-H. Wehr M. C. Rossner M. J. (2026). Revitalizing psychopharmacology in the GWAS era: the potential of barcoded screening in drug discovery. Curr. Opin. Genet. Dev. 96:102425. doi: 10.1016/j.gde.2025.102425 41505808

[ref69] Helsmoortel C. Swagemakers S. M. A. Vandeweyer G. Stubbs A. P. Palli I. Mortier G. . (2016). Whole genome sequencing of a dizygotic twin suggests a role for the serotonin receptor HTR7 in autism spectrum disorder. Am. J. Med. Genet. B Neuropsychiatr. Genet. 171, 1049–1056. doi: 10.1002/ajmg.b.32473, 27380831

[ref70] Herholt A. Brankatschk B. Kannaiyan N. Papiol S. Wichert S. P. Wehr M. C. . (2018). Pathway sensor-based functional genomics screening identifies modulators of neuronal activity. Sci. Rep. 8:17597. doi: 10.1038/s41598-018-36008-9, 30514868 PMC6279925

[ref71] Hernandez-Lopez S. Tkatch T. Perez-Garci E. Galarraga E. Bargas J. Hamm H. (2000). D2 dopamine receptors in striatal medium spiny neurons reduce L-type Ca2+ currents and excitability via a novel PLC[beta]1-IP3-calcineurin-signaling cascade. J. Neurosci. 20, 8987–8995. doi:doi: 10.1523/JNEUROSCI.20-24-08987.2000, 11124974 11124974 PMC6773013

[ref72] Hodos R. Zhang P. Lee H.-C. Duan Q. Wang Z. Clark N. R. . (2018). Cell-specific prediction and application of drug-induced gene expression profiles. Pac. Symp. Biocomput. Pac. Symp. Biocomput. 23, 32–43. doi: 10.1142/9789813235533_0004 29218867 PMC5753597

[ref73] Howes O. D. Bukala B. R. Beck K. (2024). Schizophrenia: from neurochemistry to circuits, symptoms and treatments. Nat. Rev. Neurol. 20, 22–35. doi: 10.1038/s41582-023-00904-0, 38110704

[ref74] Javitt D. C. (2023). Cognitive impairment associated with schizophrenia: from pathophysiology to treatment. Annu. Rev. Pharmacol. Toxicol. 63, 119–141. doi: 10.1146/annurev-pharmtox-051921-093250, 36151052

[ref75] Jiang H. Galtes D. Wang J. Rockman H. A. (2022). G protein-coupled receptor signaling: transducers and effectors. Am. J. Physiol.-Cell Physiol. 323, C731–C748. doi: 10.1152/ajpcell.00210.2022, 35816644 PMC9448338

[ref76] John S. Jaeggi A. V. (2021). Oxytocin levels tend to be lower in autistic children: A meta-analysis of 31 studies. Autism Int. J. Res. Pract. 25, 2152–2161. doi: 10.1177/13623613211034375, 34308675

[ref77] Jones-Tabah J. Martin R. D. Chen J. J. Tanny J. C. Clarke P. B. S. Hébert T. E. (2022). A role for BET proteins in regulating basal, dopamine-induced and cAMP/PKA-dependent transcription in rat striatal neurons. Cell. Signal. 91:110226. doi: 10.1016/j.cellsig.2021.110226, 34974082

[ref78] Kahsai A. W. Shah K. S. Shim P. J. Lee M. A. Shreiber B. N. Schwalb A. M. . (2023). Signal transduction at GPCRs: allosteric activation of the ERK MAPK by β-arrestin. Proc. Natl. Acad. Sci. 120:e2303794120. doi: 10.1073/pnas.2303794120, 37844230 PMC10614829

[ref79] Kandjani O. J. Yaqoubi S. Vahdati S. S. Borhannejad B. Dastmalchi S. Alizadeh A. A. (2023). S1PR1 modulators in multiple sclerosis: efficacy, safety, comparison, and chemical structure insights. Eur. J. Med. Chem. 250:115182. doi: 10.1016/j.ejmech.2023.115182, 36758307

[ref80] Kanzato N. Nakachi K. Mochizuki S. (2024). Istradefylline/L-DOPA Parkinson’s disease therapy and energy coupling. Neurol. Clin. Neurosci. 12, 296–305. doi: 10.1111/ncn3.12820

[ref81] Kaul I. Sawchak S. Walling D. P. Tamminga C. A. Breier A. Zhu H. . (2024). Efficacy and safety of Xanomeline-Trospium chloride in schizophrenia: a randomized clinical trial. JAMA Psychiatr. 81, 749–756. doi: 10.1001/jamapsychiatry.2024.0785, 38691387 PMC11063924

[ref82] Khan M. Z. He L. (2017). Neuro-psychopharmacological perspective of orphan receptors of rhodopsin (class A) family of G protein-coupled receptors. Psychopharmacology 234, 1181–1207. doi: 10.1007/s00213-017-4586-9, 28289782

[ref83] Kheirbek M. A. Britt J. P. Beeler J. A. Ishikawa Y. McGehee D. S. Zhuang X. (2009). Adenylyl cyclase type 5 contributes to Corticostriatal plasticity and striatum-dependent learning. J. Neurosci. 29, 12115–12124. doi: 10.1523/JNEUROSCI.3343-09.2009, 19793969 PMC2782774

[ref84] Khodaverdi M. Rahdar M. Davoudi S. Hajisoltani R. Tavassoli Z. Ghasemi Z. . (2021). 5-HT7 receptor activation rescues impaired synaptic plasticity in an autistic-like rat model induced by prenatal VPA exposure. Neurobiol. Learn. Mem. 183:107462. doi: 10.1016/j.nlm.2021.107462, 34015444

[ref85] Kim J. Choi C. (2024). Orphan GPCRs in neurodegenerative disorders: integrating structural biology and drug discovery approaches. Curr. Issues Mol. Biol. 46, 11646–11664. doi: 10.3390/cimb46100691, 39451571 PMC11505999

[ref86] Kim T. Y. Lee B. D. (2025). Current therapeutic strategies in Parkinson’s disease: future perspectives. Mol. Cells 48:100274. doi: 10.1016/j.mocell.2025.100274, 40914459 PMC12481064

[ref87] Kingwell K. (2024). FDA approves first schizophrenia drug with new mechanism of action since 1950s. Nat. Rev. Drug Discov. 23:803. doi: 10.1038/d41573-024-00155-8, 39333712

[ref88] Kong T. Liu M. Ji B. Bai B. Cheng B. Wang C. (2019). Role of the extracellular signal-regulated kinase 1/2 signaling pathway in ischemia-reperfusion injury. Front. Physiol. 10:1038. doi: 10.3389/fphys.2019.01038, 31474876 PMC6702336

[ref89] Kroeze W. K. Sassano M. F. Huang X.-P. Lansu K. McCorvy J. D. Giguère P. M. . (2015). PRESTO-tango as an open-source resource for interrogation of the druggable human GPCRome. Nat. Struct. Mol. Biol. 22, 362–369. doi: 10.1038/nsmb.3014, 25895059 PMC4424118

[ref90] Krumm B. E. Roth B. L. (2025). Intracellular GPCR modulators enable precision pharmacology. NPJ Drug Discov. 2:8. doi: 10.1038/s44386-025-00011-8, 40371403 PMC12069105

[ref91] Krystal J. H. Kavalali E. T. Monteggia L. M. (2024). Ketamine and rapid antidepressant action: new treatments and novel synaptic signaling mechanisms. Neuropsychopharmacology 49, 41–50. doi: 10.1038/s41386-023-01629-w, 37488280 PMC10700627

[ref92] Kwiatkowski T. J. Bosco D. A. Leclerc A. L. Tamrazian E. Vanderburg C. R. Russ C. . (2009). Mutations in the FUS/TLS gene on chromosome 16 cause familial amyotrophic lateral sclerosis. Science 323, 1205–1208. doi: 10.1126/science.1166066, 19251627

[ref93] Kwon Y. Mehta S. Clark M. Walters G. Zhong Y. Lee H. N. . (2022). Non-canonical β-adrenergic activation of ERK at endosomes. Nature 611, 173–179. doi: 10.1038/s41586-022-05343-3 36289326 PMC10031817

[ref94] Labus J. Röhrs K.-F. Ackmann J. Varbanov H. Müller F. E. Jia S. . (2021). Amelioration of tau pathology and memory deficits by targeting 5-HT7 receptor. Prog. Neurobiol. 197:101900. doi: 10.1016/j.pneurobio.2020.101900, 32841723

[ref95] Lacroix C. Guilhaumou R. Micallef J. Bruneteau G. Desnuelle C. Blin O. (2023). Cannabis for the treatment of amyotrophic lateral sclerosis: what is the patients’ view? Rev. Neurol. (Paris) 179, 967–974. doi: 10.1016/j.neurol.2023.03.018, 37460332

[ref96] Laere K. V. Casteels C. Dhollander I. Goffin K. Grachev I. Bormans G. . (2010). Widespread decrease of type 1 cannabinoid receptor availability in Huntington disease in vivo. J. Nucl. Med. 51, 1413–1417. doi: 10.2967/jnumed.110.077156, 20720046

[ref97] Langenhan T. Piao X. Monk K. R. (2016). Adhesion G protein-coupled receptors in nervous system development and disease. Nat. Rev. Neurosci. 17, 550–561. doi: 10.1038/nrn.2016.86, 27466150

[ref98] Lawrence A. D. Weeks R. A. Brooks D. J. Andrews T. C. Watkins L. H. Harding A. E. . (1998). The relationship between striatal dopamine receptor binding and cognitive performance in Huntington’s disease. Brain J. Neurol. 121, 1343–1355. doi: 10.1093/brain/121.7.1343, 9679785

[ref99] Lewash M. Kostenis E. Müller C. E. (2025). GPR17 - orphan G protein-coupled receptor with therapeutic potential. Trends Pharmacol. Sci. 46, 610–628. doi: 10.1016/j.tips.2025.05.001, 40544085

[ref100] Li S. H. Colson T.-L. L. Abd-Elrahman K. S. Ferguson S. S. G. (2021). Metabotropic glutamate receptor 2/3 activation improves motor performance and reduces pathology in heterozygous zQ175 Huntington disease mice. J. Pharmacol. Exp. Ther. 379, 74–84. doi: 10.1124/jpet.121.000735, 34330748

[ref101] Li M.-L. Hu X.-Q. Li F. Gao W.-J. (2015). Perspectives on the mGluR2/3 agonists as a therapeutic target for schizophrenia: still promising or a dead end? Prog. Neuro-Psychopharmacol. Biol. Psychiatry 60, 66–76. doi: 10.1016/j.pnpbp.2015.02.012, 25724760 PMC4426221

[ref102] Li H. Qiao Z. Xiao X. Cao X. Li Z. Liu M. . (2025). G protein-coupled receptors: A golden key to the treasure-trove of neurodegenerative diseases. Clin. Nutr. 46, 155–168. doi: 10.1016/j.clnu.2025.01.032 39933302

[ref103] Liu S. Bai T. Liu X. Zhao W. Li X. Sui Y. . (2025). Role and regulatory mechanism of GPR37 in neurological diseases. Front. Cell. Neurosci. 19:1617682. doi: 10.3389/fncel.2025.1617682, 40822851 PMC12350418

[ref104] Liu P.-P. Zheng H. Yang X.-X. Yang C.-C. Liu Y. Liu Y. (2021). Inhibition of TGF-β2-induced migration and epithelial-mesenchymal transition in ARPE-19 by sulforaphane. Int. J. Ophthalmol. 14, 973–980. doi: 10.18240/ijo.2021.07.03, 34282380 PMC8243186

[ref105] Lynch J. J. Van Vleet T. R. Mittelstadt S. W. Blomme E. A. G. (2017). Potential functional and pathological side effects related to off-target pharmacological activity. J. Pharmacol. Toxicol. Methods 87, 108–126. doi: 10.1016/j.vascn.2017.02.020 28216264

[ref106] Ma W. Warnhoff I. Stephan M. Ma X. Dehne K. Volkmann P. . (2025). TAOK2 controls synaptic plasticity and anxiety via ERK and calcium signaling. iScience 28:113712. doi: 10.1016/j.isci.2025.113712, 41210984 PMC12590003

[ref107] Madnani R. S. (2023). Alzheimer’s disease: a mini-review for the clinician. Front. Neurol. 14:1178588. doi: 10.3389/fneur.2023.1178588, 37426432 PMC10325860

[ref108] Mangiavacchi S. Wolf M. E. (2004). D1 dopamine receptor stimulation increases the rate of AMPA receptor insertion onto the surface of cultured nucleus accumbens neurons through a pathway dependent on protein kinase A. J. Neurochem. 88, 1261–1271. doi: 10.1046/j.1471-4159.2003.02248.x, 15009682

[ref109] Marsden C. D. (1990). Parkinson’s disease. Lancet 335, 948–949. doi: 10.1016/0140-6736(90)91006-V 1691427

[ref110] Marx W. Penninx B. W. J. H. Solmi M. Furukawa T. A. Firth J. Carvalho A. F. . (2023). Major depressive disorder. Nat. Rev. Dis. Primer 9:44. doi: 10.1038/s41572-023-00454-1 37620370

[ref111] Mateos-Aparicio P. Bello S. A. Rodríguez-Moreno A. (2020). Challenges in physiological phenotyping of hiPSC-derived neurons: from 2D cultures to 3D brain organoids. Front. Cell Dev. Biol. 8:797. doi: 10.3389/fcell.2020.00797, 32984317 PMC7479826

[ref112] McColgan P. Tabrizi S. J. (2018). Huntington’s disease: a clinical review. Eur. J. Neurol. 25, 24–34. doi: 10.1111/ene.13413, 28817209

[ref113] McDonald J. K. Ang S. Y. Langmead C. J. Stewart G. D. (2025). G protein-coupled receptor signaling in CNS (re)myelination. J. Neurochem. 169:e70286. doi: 10.1111/jnc.70286, 41257348

[ref114] McGinley M. P. Cohen J. A. (2021). Sphingosine 1-phosphate receptor modulators in multiple sclerosis and other conditions. Lancet Lond. Engl. 398, 1184–1194. doi: 10.1016/S0140-6736(21)00244-0 34175020

[ref115] McGregor M. M. Nelson A. B. (2019). Circuit mechanisms of Parkinson’s disease. Neuron 101, 1042–1056. doi: 10.1016/j.neuron.2019.03.004, 30897356

[ref116] Melnikov M. Sviridova A. Rogovskii V. Oleskin A. Boziki M. Bakirtzis C. . (2021). Serotoninergic system targeting in multiple sclerosis: the prospective for pathogenetic therapy. Mult. Scler. Relat. Disord. 51:102888. doi: 10.1016/j.msard.2021.102888, 33756440

[ref117] Meltzer H. Y. Gadaleta E. (2021). Contrasting typical and atypical antipsychotic drugs. Focus J. Life Long Learn. Psychiatry 19, 3–13. doi: 10.1176/appi.focus.20200051, 34483761 PMC8412155

[ref118] Milanese M. Bonifacino T. Torazza C. Provenzano F. Kumar M. Ravera S. . (2021). Blocking glutamate mGlu5 receptors with the negative allosteric modulator CTEP improves disease course in SOD1G93A mouse model of amyotrophic lateral sclerosis. Br. J. Pharmacol. 178, 3747–3764. doi: 10.1111/bph.15515, 33931856 PMC8457068

[ref119] Moraczewski J. Awosika A. O. Aedma K. K. (2023). “Tricyclic antidepressants,” in StatPearls, (StatPearls Publishing). 32491723

[ref120] Morano A. Fanella M. Albini M. Cifelli P. Palma E. Giallonardo A. T. . (2020). Cannabinoids in the treatment of epilepsy: current status and future prospects. Neuropsychiatr. Dis. Treat. 16, 381–396. doi: 10.2147/NDT.S203782, 32103958 PMC7012327

[ref121] Mukherjee S. Manahan-Vaughan D. (2013). Role of metabotropic glutamate receptors in persistent forms of hippocampal plasticity and learning. Neuropharmacology 66, 65–81. doi: 10.1016/j.neuropharm.2012.06.005, 22743159

[ref122] Najm F. J. Madhavan M. Zaremba A. Shick E. Karl R. T. Factor D. C. . (2015). Drug-based modulation of endogenous stem cells promotes functional remyelination in vivo. Nature 522, 216–220. doi: 10.1038/nature14335, 25896324 PMC4528969

[ref123] Nakanishi S. (1994). Metabotropic glutamate receptors: synaptic transmission, modulation, and plasticity. Neuron 13, 1031–1037. doi: 10.1016/0896-6273(94)90043-4, 7946343

[ref124] Neumann M. Sampathu D. M. Kwong L. K. Truax A. C. Micsenyi M. C. Chou T. T. . (2006). Ubiquitinated TDP-43 in frontotemporal lobar degeneration and amyotrophic lateral sclerosis. Science 314, 130–133. doi: 10.1126/science.1134108 17023659

[ref125] Nichols E. Steinmetz J. D. Vollset S. E. Fukutaki K. Chalek J. Abd-Allah F. . (2022). Estimation of the global prevalence of dementia in 2019 and forecasted prevalence in 2050: an analysis for the global burden of disease study 2019. Lancet Public Health 7, e105–e125. doi: 10.1016/S2468-2667(21)00249-8, 34998485 PMC8810394

[ref126] Nielsen B. E. Ford C. P. (2024). Reduced striatal M4-cholinergic signaling following dopamine loss contributes to parkinsonian and l-DOPA–induced dyskinetic behaviors. Sci. Adv. 10:eadp6301. doi: 10.1126/sciadv.adp6301, 39565858 PMC11578179

[ref127] Nojimoto F. D. Mueller A. Hebeler-Barbosa F. Akinaga J. Lima V. Kiguti L. R. A. . (2010). The tricyclic antidepressants amitriptyline, nortriptyline and imipramine are weak antagonists of human and rat α1B-adrenoceptors. Neuropharmacology 59, 49–57. doi: 10.1016/j.neuropharm.2010.03.015, 20363235

[ref128] Nouh R. A. Kamal A. Abdelnaser A. (2023). Cannabinoids and multiple sclerosis: A critical analysis of therapeutic potentials and safety concerns. Pharmaceutics 15:1151. doi: 10.3390/pharmaceutics15041151, 37111637 PMC10146800

[ref129] O’Connell K. S. Koromina M. van der Veen T. Boltz T. David F. S. Yang J. M. K. . (2025). Genomics yields biological and phenotypic insights into bipolar disorder. Nature 639, 968–975. doi: 10.1038/s41586-024-08468-9 39843750 PMC12163093

[ref130] Obara K. Usami Y. Okamoto R. Yoshioka K. Tanaka Y. (2024). Evaluation of inhibitory actions of antidepressants on muscarinic receptors assessed by a binding assay in the mouse cerebral neocortex. J. Pharmacol. Sci. 156, 214–217. doi: 10.1016/j.jphs.2024.09.001, 39608845

[ref131] Oblak A. Gibbs T. T. Blatt G. J. (2013). Reduced serotonin receptor subtypes in a limbic and a neocortical region in autism. Autism Res. 6, 571–583. doi: 10.1002/aur.1317 23894004 PMC3859849

[ref132] Oliva V. Fico G. Prisco M. D. Gonda X. Rosa A. R. Vieta E. (2025). Bipolar disorders: an update on critical aspects. Lancet Reg. Health – Eur. 48:101135. doi: 10.1016/j.lanepe.2024.101135, 39811787 PMC11732062

[ref133] Olmedo-Saura G. Bernardi E. Bojtos L. Martínez-Horta S. Pagonabarraga J. Kulisevsky J. . (2025). Update on the symptomatic treatment of Huntington’s disease: from pathophysiology to clinical practice. Int. J. Mol. Sci. 26:6220. doi: 10.3390/ijms26136220, 40650011 PMC12250415

[ref134] Olson P. A. Tkatch T. Hernandez-Lopez S. Ulrich S. Ilijic E. Mugnaini E. . (2005). G-protein-coupled receptor modulation of striatal CaV1.3 L-type Ca2+ channels is dependent on a shank-binding domain. J. Neurosci. 25, 1050–1062. doi: 10.1523/JNEUROSCI.3327-04.2005, 15689540 PMC6725968

[ref135] Onakpoya I. J. Heneghan C. J. Aronson J. K. (2016). Post-marketing withdrawal of 462 medicinal products because of adverse drug reactions: A systematic review of the world literature. BMC Med. 14:10. doi: 10.1186/s12916-016-0553-2, 26843061 PMC4740994

[ref136] Owen M. J. Bray N. J. Walters J. T. R. O’Donovan M. C. (2025). Genomics of schizophrenia, bipolar disorder and major depressive disorder. Nat. Rev. Genet. 26, 862–877. doi: 10.1038/s41576-025-00843-0 40355602

[ref137] Owen M. J. Legge S. E. Rees E. Walters J. T. R. O’Donovan M. C. (2023). Genomic findings in schizophrenia and their implications. Mol. Psychiatry 28, 3638–3647. doi: 10.1038/s41380-023-02293-8, 37853064 PMC10730422

[ref138] Öz-Arslan D. Yavuz M. Kan B. (2024). Exploring orphan GPCRs in neurodegenerative diseases. Front. Pharmacol. 15:1394516. doi: 10.3389/fphar.2024.1394516, 38895631 PMC11183337

[ref139] Pagano G. Niccolini F. Politis M. (2016). Current status of PET imaging in Huntington’s disease. Eur. J. Nucl. Med. Mol. Imaging 43, 1171–1182. doi: 10.1007/s00259-016-3324-6, 26899245 PMC4844650

[ref140] Pakharukova N. Masoudi A. Pani B. Staus D. P. Lefkowitz R. J. (2020). Allosteric activation of proto-oncogene kinase Src by GPCR–beta-arrestin complexes. J. Biol. Chem. 295, 16773–16784. doi: 10.1074/jbc.RA120.015400, 32978252 PMC7864071

[ref141] Patti F. Messina S. Solaro C. Amato M. P. Bergamaschi R. Bonavita S. . (2016). Efficacy and safety of cannabinoid oromucosal spray for multiple sclerosis spasticity. J. Neurol. Neurosurg. Psychiatry 87, 944–951. doi: 10.1136/jnnp-2015-312591, 27160523 PMC5013116

[ref142] Pavese N. Andrews T. C. Brooks D. J. Ho A. K. Rosser A. E. Barker R. A. . (2003). Progressive striatal and cortical dopamine receptor dysfunction in Huntington’s disease: a PET study. Brain J. Neurol. 126, 1127–1135. doi: 10.1093/brain/awg119, 12690052

[ref143] Popović L. Brankatschk B. Palladino G. Rossner M. J. Wehr M. C. (2024a). Polypharmacological profiling across protein target families and cellular pathways using the multiplexed cell-based assay platform safetyProfiler reveals efficacy, potency and side effects of drugs. Biomed. Pharmacother. Biomedecine Pharmacother. 180:117523. doi: 10.1016/j.biopha.2024.117523 39405910

[ref144] Popović L. Rossner M. J. Wehr M. C. (2024b). Protocol for identifying properties of ERBB receptor antagonists using the barcoded ERBBprofiler assay. STAR Protoc. 5:102987. doi: 10.1016/j.xpro.2024.102987, 38635397 PMC11043852

[ref145] Popović L. Wintgens J. P. Wu Y. Brankatschk B. Menninger S. Degenhart C. . (2024c). Profiling of ERBB receptors and downstream pathways reveals selectivity and hidden properties of ERBB4 antagonists. iScience 27:108839. doi: 10.1016/j.isci.2024.108839, 38303712 PMC10831936

[ref146] Prasad K. de Vries E. F. J. Elsinga P. H. Dierckx R. A. J. O. van Waarde A. (2021). Allosteric interactions between adenosine A2A and dopamine D2 receptors in Heteromeric complexes: biochemical and pharmacological characteristics, and opportunities for PET imaging. Int. J. Mol. Sci. 22:1719. doi: 10.3390/ijms22041719, 33572077 PMC7915359

[ref147] Qian H. Shu C. Xiao L. Wang G. (2022). Histamine and histamine receptors: roles in major depressive disorder. Front. Psych. 13:825591. doi: 10.3389/fpsyt.2022.825591, 36213905 PMC9537353

[ref148] Rahman M. M. Islam M. R. Mim S. A. Sultana N. Chellappan D. K. Dua K. . (2022). Insights into the promising Prospect of G protein and GPCR-mediated signaling in Neuropathophysiology and its therapeutic regulation. Oxidative Med. Cell. Longev. 2022:8425640. doi: 10.1155/2022/8425640, 36187336 PMC9519337

[ref149] Rai S. N. Dilnashin H. Birla H. Singh S. S. Zahra W. Rathore A. S. . (2019). The role of PI3K/Akt and ERK in neurodegenerative disorders. Neurotox. Res. 35, 775–795. doi: 10.1007/s12640-019-0003-y, 30707354

[ref150] Ran Y. Niu X.-N. Wang Y.-J. Xu W.-H. Liang J.-H. Xu Y. . (2025). Activation of S1PR1 by Ponesimod for multiple sclerosis therapy: uncovering MAPK and PI3K pathway mechanisms and repurposing potential. Chem. Eur. J. 31:e04742. doi: 10.1002/chem.202404742 40708468

[ref151] Rawson N. S. B. (2016). Drug safety: withdrawn medications are only part of the picture. BMC Med. 14:28. doi: 10.1186/s12916-016-0579-5, 26873482 PMC4752784

[ref152] Rendón-Ochoa E. A. Padilla-Orozco M. Calderon V. M. Avilés-Rosas V. H. Hernández-González O. Hernández-Flores T. . (2022). Dopamine D2 and adenosine A2A receptors interaction on Ca2+ current modulation in a rodent model of parkinsonism. ASN Neuro 14:17590914221102075. doi: 10.1177/17590914221102075, 36050845 PMC9178983

[ref153] Rengasamy M. Mathew S. Howland R. Griffo A. Panny B. Price R. (2024). Neural connectivity moderators and mechanisms of ketamine treatment among treatment-resistant depressed patients: a randomized controlled trial. EBioMedicine 99:104902. doi: 10.1016/j.ebiom.2023.104902, 38141395 PMC10788398

[ref154] Reul J. M. H. M. Holsboer F. (2002). On the role of corticotropin-releasing hormone receptors in anxiety and depression. Dialogues Clin. Neurosci. 4, 31–46. doi: 10.31887/DCNS.2002.4.1/jreul, 22033745 PMC3181666

[ref155] Rifkin R. A. Moss S. J. Slesinger P. A. (2017). G Protein-Gated Potassium Channels: A Link to Drug Addiction. Trends Pharmacol. Sci., 378–392. doi: 10.1016/j.tips.2017.01.007 28188005 PMC5368012

[ref156] Roher A. E. Lowenson J. D. Clarke S. Woods A. S. Cotter R. J. Gowing E. . (1993). beta-amyloid-(1-42) is a major component of cerebrovascular amyloid deposits: implications for the pathology of Alzheimer disease. Proc. Natl. Acad. Sci. USA 90, 10836–10840. doi: 10.1073/pnas.90.22.10836, 8248178 PMC47873

[ref157] Rosen D. R. Siddique T. Patterson D. Figlewicz D. A. Sapp P. Hentati A. . (1993). Mutations in cu/Zn superoxide dismutase gene are associated with familial amyotrophic lateral sclerosis. Nature 362, 59–62. doi: 10.1038/362059a0 8446170

[ref158] Ross G. W. Petrovitch H. Abbott R. D. Nelson J. Markesbery W. Davis D. . (2004). Parkinsonian signs and substantia nigra neuron density in decendents elders without PD. Ann. Neurol. 56, 532–539. doi: 10.1002/ana.20226, 15389895

[ref159] Roth B. L. Sheffler D. J. Kroeze W. K. (2004). Magic shotguns versus magic bullets: selectively non-selective drugs for mood disorders and schizophrenia. Nat. Rev. Drug Discov. 3, 353–359. doi: 10.1038/nrd1346, 15060530

[ref160] Rylaarsdam L. Guemez-Gamboa A. (2019). Genetic causes and modifiers of autism Spectrum disorder. Front. Cell. Neurosci. 13:385. doi: 10.3389/fncel.2019.00385, 31481879 PMC6710438

[ref161] Sadler F. Ma N. Ritt M. Sharma Y. Vaidehi N. Sivaramakrishnan S. (2023). Autoregulation of GPCR signalling through the third intracellular loop. Nature 615, 734–741. doi: 10.1038/s41586-023-05789-z, 36890236 PMC10033409

[ref162] Saito A. Kise R. Inoue A. (2024). Generation of comprehensive GPCR-transducer-deficient cell lines to dissect the complexity of GPCR signaling. Pharmacol. Rev. 76, 599–619. doi: 10.1124/pharmrev.124.001186, 38719480

[ref163] Sammaraiee Y. Yardley M. Keenan L. Buchanan K. Stevenson V. Farrell R. (2019). Intrathecal baclofen for multiple sclerosis related spasticity: A twenty year experience. Mult. Scler. Relat. Disord. 27, 95–100. doi: 10.1016/j.msard.2018.10.009, 30366276

[ref164] San Hernandez A. M. Singh C. Valero D. J. Nisar J. Trujillo Ramirez J. I. Kothari K. K. . (2020). Multiple sclerosis and serotonin: potential therapeutic applications. Cureus 12:e11293. doi: 10.7759/cureus.11293, 33274166 PMC7707915

[ref165] Sanchez-Vives M. V. Barbero-Castillo A. Perez-Zabalza M. Reig R. (2021). GABAB receptors: modulation of thalamocortical dynamics and synaptic plasticity. Neuroscience 456, 131–142. doi: 10.1016/j.neuroscience.2020.03.011, 32194227

[ref166] Sandrin V. Wörsdörfer B. Vardy S. Coopersmith E. Stahl M. (2024). Making good decisions in early drug discovery. Drug Discov. Today 29:104016. doi: 10.1016/j.drudis.2024.104016, 38719144

[ref167] Scheltens P. De Strooper B. Kivipelto M. Holstege H. Chételat G. Teunissen C. E. . (2021). Alzheimer’s disease, Alzheimer's disease. Lancet Lond. Engl 397, 1577–1590. doi: 10.1016/S0140-6736(20)32205-4, PMC8354300 33667416

[ref168] Schiöth H. B. Fredriksson R. (2005). The GRAFS classification system of G-protein coupled receptors in comparative perspective. Gen. Comp. Endocrinol. 142, 94–101. doi: 10.1016/j.ygcen.2004.12.018, 15862553

[ref169] Schuhmacher A. Hinder M. Brief E. Gassmann O. Hartl D. (2025). Benchmarking R&D success rates of leading pharmaceutical companies: an empirical analysis of FDA approvals (2006–2022). Drug Discov. Today 30:104291. doi: 10.1016/j.drudis.2025.104291, 39805539

[ref170] Secher A. Jelsing J. Baquero A. F. Hecksher-Sørensen J. Cowley M. A. Dalbøge L. S. . (2014). The arcuate nucleus mediates GLP-1 receptor agonist liraglutide-dependent weight loss. J. Clin. Invest. 124, 4473–4488. doi: 10.1172/JCI75276, 25202980 PMC4215190

[ref171] Seo J. Kritskiy O. Watson L. A. Barker S. J. Dey D. Raja W. K. . (2017). Inhibition of p25/Cdk5 attenuates Tauopathy in mouse and iPSC models of frontotemporal dementia. J. Neurosci. 37, 9917–9924. doi: 10.1523/JNEUROSCI.0621-17.2017, 28912154 PMC5637118

[ref172] Shukla A. K. Westfield G. H. Xiao K. Reis R. I. Huang L.-Y. Tripathi-Shukla P. . (2014). Visualization of arrestin recruitment by a G-protein-coupled receptor. Nature 512, 218–222. doi: 10.1038/nature13430, 25043026 PMC4134437

[ref173] Sorosina M. Clarelli F. Ferrè L. Osiceanu A. M. Unal N. T. Mascia E. . (2018). Clinical response to Nabiximols correlates with the downregulation of immune pathways in multiple sclerosis. Eur. J. Neurol. 25, 934–e70. doi: 10.1111/ene.13623 29528549

[ref174] Stamoula E. Siafis S. Dardalas I. Ainatzoglou A. Matsas A. Athanasiadis T. . (2021). Antidepressants on multiple sclerosis: A review of in vitro and in vivo models. Front. Immunol. 12:677879. doi: 10.3389/fimmu.2021.677879, 34093579 PMC8173210

[ref175] Stavrogianni K. Kitsos D. K. Giannopapas V. Smyrni V. Chasiotis A. K. Akrivaki A. . (2025). Evaluating vaporized cannabinoid therapy in multiple sclerosis: findings from a prospective single-center clinical study. J. Clin. Med. 14:2121. doi: 10.3390/jcm14062121, 40142928 PMC11943353

[ref176] Suh Y. H. Chang K. Roche K. W. (2018). Metabotropic glutamate receptor trafficking. Mol. Cell. Neurosci. 91, 10–24. doi: 10.1016/j.mcn.2018.03.014, 29604330 PMC6128748

[ref177] Sun D. Gao W. Hu H. Zhou S. (2022). Why 90% of clinical drug development fails and how to improve it? Acta Pharm. Sin. B 12, 3049–3062. doi: 10.1016/j.apsb.2022.02.002, 35865092 PMC9293739

[ref178] Surmeier D. J. Bargas J. Hemmings H. C. Nairn A. C. Greengard P. (1995). Modulation of calcium currents by a D1 dopaminergic protein kinase/phosphatase cascade in rat neostriatal neurons. Neuron 14, 385–397. doi: 10.1016/0896-6273(95)90294-5, 7531987

[ref179] Surmeier D. J. Ding J. Day M. Wang Z. Shen W. (2007). D1 and D2 dopamine-receptor modulation of striatal glutamatergic signaling in striatal medium spiny neurons. Trends Neurosci. 30, 228–235. doi: 10.1016/j.tins.2007.03.008, 17408758

[ref180] Suzuki N. Hajicek N. Kozasa T. (2009). Regulation and physiological functions of G12/13-mediated signaling pathways. Neurosignals 17, 55–70. doi: 10.1159/000186690, 19212140 PMC2836950

[ref181] Svensson K. A. Hao J. Bruns R. F. (2019). Positive allosteric modulators of the dopamine D1 receptor: A new mechanism for the treatment of neuropsychiatric disorders. Adv. Pharmacol. San Diego Calif 86, 273–305. doi: 10.1016/bs.apha.2019.06.001 31378255

[ref182] Tabrizi S. J. Schobel S. Gantman E. C. Mansbach A. Borowsky B. Konstantinova P. . (2022). A biological classification of Huntington’s disease: the integrated staging system. Lancet Neurol. 21, 632–644. doi: 10.1016/S1474-4422(22)00120-X, 35716693

[ref183] Tamagno E. Guglielmotto M. Giliberto L. Vitali A. Borghi R. Autelli R. . (2009). JNK and ERK1/2 pathways have a dual opposite effect on the expression of BACE1. Neurobiol. Aging 30, 1563–1573. doi: 10.1016/j.neurobiolaging.2007.12.015, 18255190

[ref184] Trauernicht M. Filipovska T. Rastogi C. van Steensel B. (2024). Optimized reporters for multiplexed detection of transcription factor activity. Cell Syst. 15, 1107–1122.e7. doi: 10.1016/j.cels.2024.11.003, 39644900 PMC11667439

[ref185] Trubetskoy V. Pardiñas A. F. Qi T. Panagiotaropoulou G. Awasthi S. Bigdeli T. B. . (2022). Mapping genomic loci implicates genes and synaptic biology in schizophrenia. Nature 604, 502–508. doi: 10.1038/s41586-022-04434-5, 35396580 PMC9392466

[ref186] Tsien R. W. Rosenberg E. C. (2025). Ion channels and G protein-coupled receptors: Cannabidiol actions on disorders of excitability and synaptic excitatory-inhibitory ratio. Mol. Pharmacol. 107:100017. doi: 10.1016/j.molpha.2025.100017, 40048808 PMC13095461

[ref187] Tsuga H. Okuno E. Kameyama K. Haga T. (1998). Sequestration of human muscarinic acetylcholine receptor hm1-hm5 subtypes: effect of G protein-coupled receptor kinases GRK2, GRK4, GRK5 and GRK6. J. Pharmacol. Exp. Ther. 284, 1218–1226. doi: 10.1016/S0022-3565(24)37343-4, 9495886

[ref188] Turri M. Teatini F. Donato F. Zanette G. Tugnoli V. Deotto L. . (2018). Pain modulation after Oromucosal cannabinoid spray (SATIVEX®) in patients with multiple sclerosis: A study with quantitative sensory testing and laser-evoked potentials. Med. Basel Switz. 5:59. doi: 10.3390/medicines5030059, 29933552 PMC6163235

[ref189] Uhlén M. Fagerberg L. Hallström B. M. Lindskog C. Oksvold P. Mardinoglu A. . (2015). Proteomics. Tissue-based map of the human proteome. Science 347:1260419. doi: 10.1126/science.1260419, 25613900

[ref190] van der Poel M. Ulas T. Mizee M. R. Hsiao C.-C. Miedema S. S. M. Adelia N. . (2019). Transcriptional profiling of human microglia reveals grey–white matter heterogeneity and multiple sclerosis-associated changes. Nat. Commun. 10:1139. doi: 10.1038/s41467-019-08976-7, 30867424 PMC6416318

[ref191] Volonté C. Apolloni S. Amadio S. (2022). “The histamine and multiple sclerosis Alliance: pleiotropic actions and functional validation,” in The Functional Roles of Histamine Receptors, eds. Yanai K. Passani M. B. (Cham: Springer International Publishing), 217–239. 10.1007/7854_2021_240 34432258

[ref192] Walker F. O. (2007). Huntington’s disease. Lancet 369, 218–228. doi: 10.1016/S0140-6736(07)60111-1 17240289

[ref193] Walsh G. M. (2002). Emerging safety issues regarding long-term usage of H(1)receptor antagonists. Expert Opin. Drug Saf. 1, 225–235. doi: 10.1517/14740338.1.3.225, 12904138

[ref194] Wang H. Xu J. Lazarovici P. Quirion R. Zheng W. (2018). cAMP response element-binding protein (CREB): A possible signaling molecule link in the pathophysiology of schizophrenia. Front. Mol. Neurosci. 11:255. doi: 10.3389/fnmol.2018.00255, 30214393 PMC6125665

[ref195] Wong T.-S. Li G. Li S. Gao W. Chen G. Gan S. . (2023). G protein-coupled receptors in neurodegenerative diseases and psychiatric disorders. Signal Transduct. Target. Ther. 8:177. doi: 10.1038/s41392-023-01427-2, 37137892 PMC10154768

[ref196] Wu Y. Jensen N. Rossner M. J. Wehr M. C. (2024). Exploiting cell-based assays to accelerate drug development for G protein-coupled receptors. Int. J. Mol. Sci. 25:5474. doi: 10.3390/ijms25105474, 38791511 PMC11121687

[ref197] Xu Y. Zhi F. Balboni G. Yang Y. Xia Y. (2020). Opposite roles of δ- and μ-opioid receptors in BACE1 regulation and Alzheimer’s injury. Front. Cell. Neurosci. 14:88. doi: 10.3389/fncel.2020.00088, 32425755 PMC7204847

[ref198] Yang Y. Tian X. Xu D. Zheng F. Lu X. Zhang Y. . (2018). GPR40 modulates epileptic seizure and NMDA receptor function. Sci. Adv. 4:eaau2357. doi: 10.1126/sciadv.aau2357, 30345361 PMC6192686

[ref199] Yang H. Wang Y. Liu W. He T. Liao J. Qian Z. . (2024). Genome-wide pan-GPCR cell libraries accelerate drug discovery. Acta Pharm. Sin. B 14, 4296–4311. doi: 10.1016/j.apsb.2024.06.023, 39525595 PMC11544303

[ref200] Yang D. Zhou Q. Labroska V. Qin S. Darbalaei S. Wu Y. . (2021). G protein-coupled receptors: structure- and function-based drug discovery. Signal Transduct. Target. Ther. 6:7. doi: 10.1038/s41392-020-00435-w, 33414387 PMC7790836

[ref201] Young Yang M. Duy Mac K. Strzelinski H. R. Hoffman S. A. Kim D. Kim S.-K. . (2024). Agonist activation to open the gα subunit of the GPCR–G protein precoupled complex defines functional agonist activation of TAS2R5. Proc. Natl. Acad. Sci. 121:e2409987121. doi: 10.1073/pnas.2409987121, 39565310 PMC11621838

[ref202] Yu Y. Nguyen D. T. Jiang J. (2019). G protein-coupled receptors in acquired epilepsy: Druggability and translatability. Prog. Neurobiol. 183:101682. doi: 10.1016/j.pneurobio.2019.101682, 31454545 PMC6927250

[ref203] Yuan Y. Chen J. Ge X. Deng J. Xu X. Zhao Y. . (2021). Activation of ERK-Drp1 signaling promotes hypoxia-induced aβ accumulation by upregulating mitochondrial fission and BACE1 activity. FEBS Open Bio 11, 2740–2755. doi: 10.1002/2211-5463.13273, 34403210 PMC8487051

[ref204] Zahm A. M. Owens W. S. Himes S. R. Fallon B. S. Rondem K. E. Gormick A. N. . (2024). A massively parallel reporter assay library to screen short synthetic promoters in mammalian cells. Nat. Commun. 15:10353. doi: 10.1038/s41467-024-54502-9, 39609378 PMC11604768

[ref205] Zeghal M. Laroche G. Freitas J. D. Wang R. Giguère P. M. (2023). Profiling of basal and ligand-dependent GPCR activities by means of a polyvalent cell-based high-throughput platform. Nat. Commun. 14:3684. doi: 10.1038/s41467-023-39132-x, 37407564 PMC10322906

[ref206] Zeidan J. Fombonne E. Scorah J. Ibrahim A. Durkin M. S. Saxena S. . (2022). Global prevalence of autism: A systematic review update. *Autism res*. Off. J. Int. Soc. Autism Res. 15, 778–790. doi: 10.1002/aur.2696, 35238171 PMC9310578

[ref207] Zhang M. Chen T. Lu X. Lan X. Chen Z. Lu S. (2024). G protein-coupled receptors (GPCRs): advances in structures, mechanisms and drug discovery. Signal Transduct. Target. Ther. 9:88. doi: 10.1038/s41392-024-01803-6, 38594257 PMC11004190

[ref208] Zhang J. Ko S.-Y. Liao Y. Kwon Y. Jeon S. J. Sohn A. . (2018). Activation of the dopamine D1 receptor can extend long-term spatial memory persistence via PKA signaling in mice. Neurobiol. Learn. Mem. 155, 568–577. doi: 10.1016/j.nlm.2018.05.016, 29803941

[ref209] Zhao J. Li X. Chen X. Cai Y. Wang Y. Sun W. . (2019). GRK5 influences the phosphorylation of tau via GSK3β and contributes to Alzheimer’s disease. J. Cell. Physiol. 234, 10411–10420. doi: 10.1002/jcp.27709, 30511419

[ref210] Zheng S. Sheng R. (2024). The emerging understanding of frizzled receptors. FEBS Lett. 598, 1939–1954. doi: 10.1002/1873-3468.14903, 38744670

[ref211] Zheng Y. VanDusen N. J. (2023). Massively parallel reporter assays for high-throughput in vivo analysis of Cis-regulatory elements. J. Cardiovasc. Dev. Dis. 10:144. doi: 10.3390/jcdd10040144, 37103023 PMC10146671

[ref212] Zhou P. Homberg J. R. Fang Q. Wang J. Li W. Meng X. . (2019). Histamine-4 receptor antagonist JNJ7777120 inhibits pro-inflammatory microglia and prevents the progression of Parkinson-like pathology and behaviour in a rat model. Brain Behav. Immun. 76, 61–73. doi: 10.1016/j.bbi.2018.11.006, 30408497

[ref213] Züchner T. Perez-Polo J. R. Schliebs R. (2004). Beta-secretase BACE1 is differentially controlled through muscarinic acetylcholine receptor signaling. J. Neurosci. Res. 77, 250–257. doi: 10.1002/jnr.20152, 15211591

